# Dysregulated lncRNAs regulate human umbilical cord mesenchymal stem cell differentiation into insulin-producing cells by forming a regulatory network with mRNAs

**DOI:** 10.1186/s13287-023-03572-5

**Published:** 2024-01-25

**Authors:** Tianqin Xie, Qiming Huang, Qiulan Huang, Yanting Huang, Shuang Liu, Haixia Zeng, Jianping Liu

**Affiliations:** 1 Department of Endocrinology Medicine, The Second Affiliated Hospital of Nanchang University, No. 1 Minde Road, Nanchang of Jiangxi, 330006 China; 2 The National Engineering Research Center for Bioengineering Drugs and the Technologies, Institute of Translation Medicine, Nanchang University, Nanchang of Jiangxi, China

**Keywords:** Umbilical cord mesenchymal stem cells, Insulin-producing cells, Long noncoding RNA, Small molecule, Diabetes mellitus

## Abstract

**Objective:**

In recent years, cell therapy has emerged as a new research direction in the treatment of diabetes. However, the underlying molecular mechanisms of mesenchymal stem cell (MSC) differentiation necessary to form such treatment have not been clarified.

**Methods:**

In this study, human umbilical cord mesenchymal stem cells (HUC-MSCs) isolated from newborns were progressively induced into insulin-producing cells (IPCs) using small molecules. HUC-MSC (S0) and four induced stage (S1–S4) samples were prepared. We then performed transcriptome sequencing experiments to obtain the dynamic expression profiles of both mRNAs and long noncoding RNAs (lncRNAs).

**Results:**

We found that the number of differentially expressed lncRNAs and mRNAs trended downwards during differentiation. Gene Ontology (GO) analysis showed that the target genes of differentially expressed lncRNAs were associated with translation, cell adhesion, and cell connection. Kyoto Encyclopedia of Genes and Genomes (KEGG) analysis revealed that the NF-KB signalling pathway, MAPK signalling pathway, HIPPO signalling pathway, PI3K–Akt signalling pathway, and p53 signalling pathway were enriched in these differentially expressed lncRNA-targeting genes. We also found that the coexpression of the lncRNA CTBP1-AS2 with PROX1 and the lncRNAs AC009014.3 and GS1-72M22.1 with JARID2 mRNA was related to the development of pancreatic beta cells. Moreover, the coexpression of the lncRNAs: XLOC_ 050969, LINC00883, XLOC_050981, XLOC_050925, MAP3K14- AS1, RP11-148K1.12, and CTD2020K17.3 with p53, regulated insulin secretion by pancreatic beta cells.

**Conclusion:**

In this study, HUC-MSCs combined with small molecule compounds were successfully induced into IPCs. Differentially expressed lncRNAs may regulate the insulin secretion of pancreatic beta cells by regulating multiple signalling pathways. The lncRNAs AC009014.3, Gs1-72m21.1, and CTBP1-AS2 may be involved in the development of pancreatic beta cells, and the lncRNAs: XLOC_050969, LINC00883, XLOC_050981, XLOC_050925, MAP3K14-AS1, RP11-148K1.12, and CTD2020K17.3 may be involved in regulating the insulin secretion of pancreatic beta cells, thus providing a lncRNA catalogue for future research regarding the mechanism of the transdifferentiation of HUC-MSCs into IPCs. It also provides a new theoretical basis for the transplantation of insulin-producing cells into diabetic patients in the future.

**Graphical Abstract:**

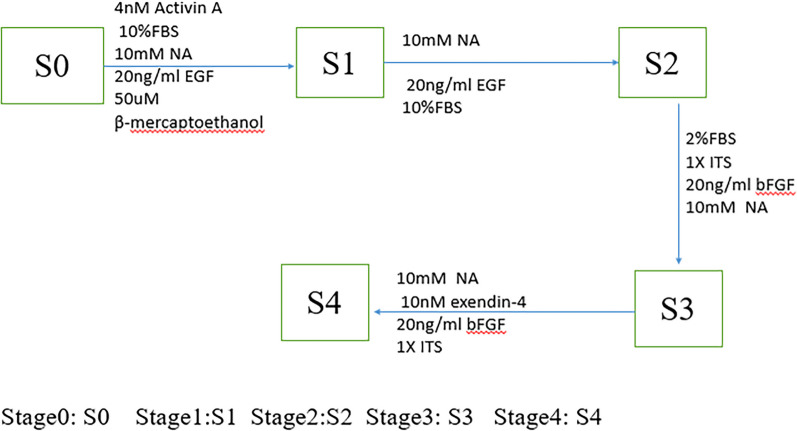

**Supplementary Information:**

The online version contains supplementary material available at 10.1186/s13287-023-03572-5.

## Introduction

Diabetes mellitus is caused by insulin secretion deficiency associated with varying degrees of damage to pancreatic beta cells, and the inability to regulate the metabolic balance of blood sugar. Therefore, replacement therapy with pancreatic beta cells has become a therapeutic option for diabetes. The clinical application of islet transplantation is limited by insufficient donors, immune rejection, immunosuppressive drug side effects, and possible beta cell toxicity [1]. Stem cells are an important alternative that could provide innumerable potential islet cell sources for transplants. Umbilical cord mesenchymal stem cells (UC-MSCs) are obtained from discarded placentas, and they are more readily available and have a higher proliferative potential than other MSCs [2, 3]. UC-MSCs are more primitive MSCs than bone marrow or adipose mesenchymal stem cells (BM-MSCs or AD-MSCs) and do not express major histocompatibility complex (MHC) class II (HLA-DR) antigens [3, 4], which makes them good candidates for potential allogeneic therapeutic applications. Previous studies have shown that UC-MSCs can differentiate into IPCs in vitro and improve the glycaemic status of diabetic mice after transplantation in vivo [5, 6]; however, the induction efficiency was low and the maintenance time of hypoglycaemia was slightly short [5–7]. Therefore, the differentiation efficiency and function of IPCs must be improved. Moreover, studies should focus on identifying the underlying molecular mechanisms by which UC-MSCs differentiate into IPCs. Many small molecule compounds, growth factors, activators, and inhibitors can transdifferentiate MSCs into IPCs, which improves the survival of IPCs and the ability to release insulin in vitro [8–11]. MSC transdifferentiation efficiency and insulin secretion can be improved by overexpression of Pdx1, Neurog3, MafA, and Pax4 [12–14]. A comprehensive understanding of the signalling pathways and temporal transcription factor activation patterns during human and rodent pancreas organogenesis has accelerated the production of IPCs from human pluripotent stem cells (hPSCs) [15–17]. Moreover, MSC differentiation is accurately regulated and coordinated by mechanical and molecular signals from extracellular environments and involves complex pathways regulated at the transcriptional and posttranscriptional levels [18–20]. Growing evidence suggests that lncRNAs are important gene expression regulators through their interactions with DNA, RNA, or proteins [21–23]. By acting as guides, scaffolds, and/or decoys, lncRNAs can modulate chromatin organization or epigenetics, regulate transcription or control mRNA progression [24]. LncRNAs play an important role in many biological processes, including development [25], differentiation [26], and metabolism [27, 28]. However, the specific mechanism by which lncRNAs regulate the transdifferentiation of MSCs into IPCs remains poorly understood. In this study, a new four-stage protocol was developed based on previous studies that investigated small molecule compounds [11]. Moreover, cells at each stage were collected for high-throughput sequencing (Illumina PE150) to obtain the expression profile of lncRNAs and mRNAs and to explore their molecular mechanisms during the differentiation of HUC-MSCs into IPCs, thereby providing potential pathways to improve the efficiency of induction in the future. To our knowledge, this is the first study to reveal the changes in lncRNA and mRNA expression during the differentiation of HUC-MSCs into IPCs, and to enrich the lncRNA gene pool for the transformation of stem cells into IPCs.

## Materials and methods

### Separation, cultivation, and identification of HUC-MSCs

Human umbilical cord (HUC) specimens were gathered from the obstetric department of the Second Affiliated Hospital of Nanchang University. The ethics committee of the Second Affiliated Hospital of Nanchang University approved this study, and informed consent was obtained from all donors. The HUC tissues were rinsed with phosphate-buffered saline (PBS). Wharton’s jelly was cut into small pieces using ophthalmic scissors and placed in a culture flask. The UC-MSCs were cultured in ḁ-MEM complete medium (Gibco, USA), containing 10% foetal bovine serum (10099-141, Gibco, USA), and 1% penicillin and streptomycin (Sigma) in a humidified atmosphere with 5% CO_2_ at 37 °C. After 5 days, the fluid was first changed, with subsequent changes every 3 days after adhesion. When the cells were 70–80% confluent, they were digested with trypsin (0.25%Trypsin, Sigma, USA) and passaged at a ratio of 1:3. The third generation was used for subsequent experiments. Uniform spindle-fibroblastic morphology and adherent phenotypes were identified using an inverted microscope (Nikon Corp., Tokyo, Japan). The third generation of UC-MSCs was suspended in PBS, stained with fluorescence-labelled monoclonal antibodies against CD34 (#343503,Biolegend, Beijing), HLA-DR (#307627, Biolegend, Beijing), CD105 (#323207, Biolegend, Beijing), CD73 (#344003, Biolegend, Beijing), CD90 (#328109, Biolegend, Beijing), CD14 (#399205, Biolegend, Beijing), CD44 (#338803, Biolegend, Beijing), and CD45 (#304025, Biolegend, Beijing) and then incubated for 30 min at 4 °C in the dark, with the inclusion of appropriate isotype controls (for each antibody isotype). The expression of these cell surface markers was evaluated using an LSRFortessa Flow Cytometer (BD). A differentiation kit (Cyagon, China) was used to identify the differentiation ability of stem cells. Briefly, UC-MSCs were counted at 2 × 10^^4^ cells/cm^2^ and seeded into a six-well plate. They were then incubated in adipose and osteoblast differentiation culture media for 14 d. Adipocytes and osteocytes were stained with oil red O and alizarin red, respectively. For chondrogenic differentiation, UC-MSCs were seeded into a density of 2 × 10^^4^ cells/cm^2^ in chondroblast differentiation culture media for 21 days. Chondrocytes were stained with Alcian blue.

### Induction of HUC-MSCs

UC-MSCs obtained from passage three were used for IPC differentiation. The differentiation protocol was adopted from previous studies [35], with slight modifications (a). After the cells were digested, they were placed into a six-well plate at a density of 2 × 10^5 cells/well in serum–free DMEM complete medium at 37 ℃in a humidified atmosphere containing 5% CO2 until cell fusion reached 70–80%. For induction, the medium was replaced with stage 1 medium containing DMEM:F12 supplemented with 10% FBS, 1% penicillin and streptomycin (Sigma), 10 mM nicotinamide (NA, Sigma), 20 ng/ml EGF (PEPROTECH,USA), 50 µM β-mercaptoethanol, and 4 nM activin A (Gibco, USA) for 3 days. On the fourth day, at stage 2, the medium was replaced with media containing the same supplements as those in the stage 1 medium, but lacking activin A and β-mercaptoethanol. The cells were then incubated for another 5 days. On the ninth day, the media were changed to stage 3 media containing DMEM:F12 (1:1) with supplemented 2% FBS, 1% penicillin and streptomycin (Sigma), 20 ng/ml bFGF (PEPROTECH,USA), 100 × ITS (#41400045, Gibco), 10 nM exendin-4 (Gibco, USA), and 10 mM NA, and the cells were cultured for another 7 d. On the sixteenth day, at stage 4, the medium was replaced with media containing the same supplements as those in stage 3, but lacking the 2% FBS supplement. The cells were allowed to mature in this medium for another 7 days. Changes in morphology were observed under a light microscope during differentiation.

### Real-time quantitative PCR (qPCR)

Total RNA was extracted at each stage using TRIzol reagent® (Tiangen Biochemical Technology, Shanghai, China) and was then reverse-transcribed into cDNA templates for qPCR amplification using PrimeScript™ RT reagent Kit with gDNA Eraser (RR047B, TaKaRa, Japan). qPCR amplification was performed using a Master cycler Realplex 2 (Eppendorf, USA), with a SYBR Green Real-Time RCR Master Mix (RR82LR, TaKaRa, Japan). The qPCR primers were synthesized by Invitrogen (Shanghai, China) and are listed in Table 1.

**Table 1:** Primer sequences used in the quantitative RT-PCR

Gene	F (5′–3′)	R (5′–3′)
FOXA2	AGCGCCCACGTACGACGACATG	AGAGCCCGAGGGCTACTCCT
Sox17	TCCCTACCAGGGACACGACT	GAGCTAGCGTCGGACACCAC
PDX1	GTCCAGCTGCCTTTCCCAT	TCCGCTTGTTCTCCTCCG
Ngn3	GGAGTCGGCGAAAGAAGG	AAGCTGTGGTCCGCTATGC
NKx6.1	TTGGACAAAGACGGGAAGAG	CTGTCATCCCCAACGAATAG
Insulin	GGGAACGAGGCTTCTTCTACA	ACAATGCCACGCTTCTGC
GLUT2	TCCAGCTACCGACAGCCTATT	GCACAAACAAACATCCCACTCA
MAFA	TTCAGCAAGGAGGAGGTCAT	CCGCCAGCTTCTCGTATTTC
GAPDH	AATCCCATCACCATCTTCCA	TGGACTCCACGACATACTCA

*F* forward primer, *R* reverse primer

### Western blot

Cells at all stages were lysed by radioimmunoprecipitation (RIPA) buffer (P0013D, Beyotime, Shanghai, China) supplemented with cocktail proteinase and phosphatase inhibitors. The protein assay was quantified with the Quantification Kit (A53225, Thermo Fisher, China). Equivalent amounts of protein were isolated by sodium dodecyl sulphate–polyacrylamide gel electrophoresis (SDS–PAGE) and wet transferred onto polyvinylidene fluoride (PVDF) membranes (IPVH00010, MILLIPORE, USA). The membranes were blocked in 5% BSA and further incubated with each primary antibody (Additional file 10: Table S2) at − 4° C overnight. Then, bands were tested by corresponding secondary antibody (Additional file 10: Table S2) and imaged through Pro-light horseradish peroxidase (HRP) chemiluminescence detection reagents (QuickChemi 5100, Monad, Beijing, China).

### In vitro insulin secretion assay

Stage IV IPCs and UC-MSCs were lightly washed twice with sugar-free KRBH buffer wash. The cells were then preincubated in KRBH culture media containing 5.5 mM or 25 mM glucose at 37 °C for 2 h, and the supernatants were then collected for insulin quantification. The insulin concentration was assessed using an ELISA kit (DINS001*KT, Alpco, Salem, USA) according to the manufacturer’s instructions. The insulin levels were calculated using a standard curve.

### Immunofluorescent

Cells from each stage of the groups described above were fixed in 4% paraformaldehyde for 20 min. After washing with PBS, the cells were permeabilized with 0.1% Triton-100 for 20 min, and then blocked with goat serum for 30 min. They were then incubated with the corresponding primary antibodies, which included Pdx1 antibody (rabbit, 1:200, #5679S, CST). C-peptide antibody (rabbit, 1:100, #4593S, CST), Foxa2 antibody (Mouse, 1:200, #ab108422,Abcam), Sox17 antibody (Mouse,1:100, #bs12205R, Bioss), MAFA antibody (rabbit, 1:200,#bs0924R, Bioss), GLUT2 antibody (rabbit, 1:200, #bs1010379R, Bioss), Nkx6.1 antibody (rabbit, 1:200, #ab221549, Abcam), and insulin antibody (rabbit, 1:200, #ab181547, Abcam) overnight at 4 ℃. Next, the cells were washed three times with PBST and further incubated for 1 h in a wet box in the dark with the following secondary antibodies: that included goat anti-rabbit immunoglobulin G heavy and light chain antibodies (IgGH&L, 1:250, Proteintech) and anti-mouse IgG (H + L), F(ab’)_2_ fragment (Alexa Fluor® 555 Conjugate). The nuclei were stained with Hoechst 33342 for 7 min after washing with PBST and were then observed under a fluorescence microscope (NOVA View^R^).

### Dithizone (DTZ) staining

A DTZ (Sigma-Aldrich, USA) stock solution was prepared by dissolving 20 mg of DTZ into 2 ml of DMSO. Stage IV IPCs were added to 1 mL of 1 × PBS buffer and 10 μL of DTZ stock solution, and this mixture was then incubated at 37 °C for 15 min. Scarlet red-stained clusters were examined under a phase-contrast microscope (Nikon Corp).

### Sample qualification and quantification

TRIzol® reagent (Invitrogen, USA) was used to extract total RNA at each stage, and genomic DNA was removed using DNase I (Takara, Japan), according to the manufacturer’s instructions. RNA samples were first subjected to 1% agarose gel electrophoresis to assess for possible contamination and degradation. A NanoPhotometer® spectrophotometer was used to examine RNA purity and concentration. The RNA Nano 6000 Assay Kit for the Bioanalyzer 2100 system was used to measure the RNA integrity and quantity.

### Library preparation

The RNA library for lncRNA-seq was prepared using rRNA depletion and the strand method. Briefly, the rRNA Removal Kit (RS-122-2402, Illumina, USA) was used to deplete ribosomal RNA from total RNA, according to the manufacturer’s instructions. RNA was then fragmented into 250–300 bp fragments, and fragmented RNA and dNTPs (dATP, dTTP, dCTP, and dGTP) were used to reverse-transcribe first-strand cDNA. RNase H was used to degrade RNA, and DNA polymerase I and dNTPs (dATP, dUTP, dCTP, and dGTP) were used to synthesize second-strand cDNA. The remaining overhangs of the double-stranded cDNA were converted into blunt ends via exonuclease/ polymerase activity. After adenylation of the 3’ ends of the DNA fragments, the sequencing connector was ligated to the cDNA. To optimize the 250–300 bp cDNA fragments, the AMPure XP system was used to purify the library fragments. Uracil-N-glycosylase was used to perform uridine digestion and followed by cDNA amplification using PCR.

After library construction, a Qubit® fluorometer was used to measure the concentration of the library; which was adjusted to 1 ng/uL. The insert size of the library was examined using an Agilent 2100 Bioanalyzer. Finally, qPCR was used to determine the accurate concentration of the cDNA library. Once the inserted library was determined to be of the same size and concentration, the sample was ready for sequencing.

### Sequencing

After library preparation and pooling of different samples, the samples were sequenced using an Illumina sequencer. Typically, lncRNA-seq uses PE150 (paired-end 150nt) sequencing to obtain 12G raw data.

#### Read cleaning process

Low-quality bases from raw reads were trimmed using the FASTX-Toolkit (v.0.013; http: // hannonlab.chsl.edu/fastx _toolkit/). The clean reads were evaluated using FastQC (http://www.bioinformatics.babraham.ac.uk/projects/fastqc).

#### Read alignment and differentially expressed gene (DEG) analysis

Clean reads were aligned to the human GRch38 genome [29] (http://support.illumina.com/sequencing/sequencing_software/igenome.html) using HISAT2 (http://ccb.jhu.edu/software/hisat2;Kim D et al. 2015). Finally, uniquely mapped reads were used to calculate the read number and fragments per kilobase of exon per million fragments mapped (FPKM) for each gene. The expression levels of the genes were evaluated by FPKM. DESeq2 software [30], which is specifically used to analyse the differential expression of genes (DEGs), was used to screen the RNA-seq data for DEGs. The results were analysed using fold change (FC ≥ 2 or ≤ 0.5) and false discovery rate (FDR ≤ 0.05) to determine whether a given gene was differentially expressed.

#### LncRNA prediction and direction identification

To systematically analyse lncRNA expression patterns, a pipeline was used for lncRNA identification in a manner similar to that used in previous studies [31], which was constructed based on Cufflinks software [32].

#### Coexpression analysis

To explore the regulatory mode between lncRNAs and their host mRNAs, Pearson’s correlation coefficients (PCCs) were calculated and the results were classified into three classes based on the PCC values: positively correlated, negatively correlated and noncorrelated.

#### K-means analysis

To identify genes that exhibited similar gene expression across different developmental stages, gene expression data were clustered using the k-means clustering algorithm as implemented in R. FPKM expression values for row-scaled genes were combined in a single matrix. To determine the appropriate group size k, k-means clustering was repeated six times with k ranging between 3 and 8.

#### Functional enrichment analysis

To sort the functional categories of DEGs, Gene Ontology (GO) terms and KEGG pathways were identified using the KOBAS 2.0 server, with enrichment of each term determined through the hypergeometric test with FDR controlled via the Benjamini–Hochberg procedure.

#### Other statistical analyses

Principal component analysis (PCA) was performed using the R package factoextra (https://cloud.r-project.org/package=factoextra) to indicate the clustering of samples with the first two components. The pheatmap package (https://cran.r-project.org/web/packages/pheatmap/index.html) in R was used to perform clustering based on Euclidean distance.

#### Statistical analysis

The experimental data were analysed and processed using the GraphPad Prism 8 system. The results are expressed as the mean ± standard deviation (*X* ± SD). The t test method was used for comparisons between two groups, and one-way analysis of variance was used for comparisons among multiple groups.

## Results

### Culture and identification of UC-MSCs

UC-MSCs appear as fibroblast spindle-like and adherent colonies when cultured in vitro (Additional file 1: Fig S1 A). The phenotype of UC-MSCs was consistent with the minimal criteria of the International Society for Cellular Therapy from 2006 [34]. Flow cytometric analysis of UC-MSCs showed that they were negative for the haematopoietic markers CD34, and HLA-DR, the leukocyte common antigen CD45, and the monocyte marker CD14, but positive for CD73, CD90, CD105, and CD44 (Additional file 1: Fig S1 B). In addition, under lineage-specific differentiation chondrocytes were observed in vitro. Alizarin red staining demonstrated the formation of mineralized nodules upon osteogenic induction. Oil red O staining showed the accumulation of lipid droplets in the cells after adipogenesis. The deposition of sulphated proteoglycans, as indicated by Alcian blue staining, demonstrated successful differentiation into chondrocytes (Additional file 1: Fig S1 C).

### In vitro IPC differentiation and dithizone (DTZ) staining

**Fig. 1:**
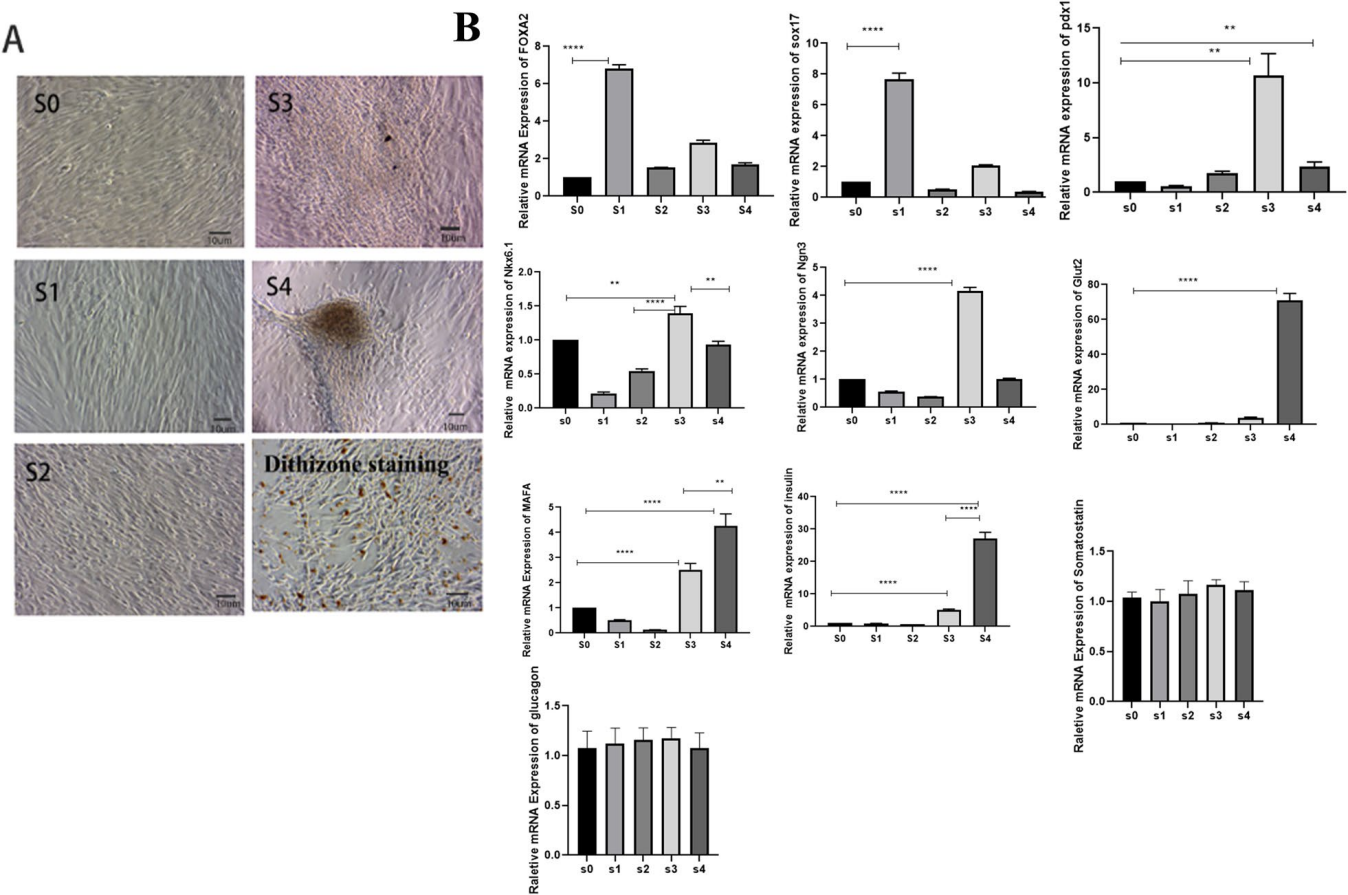
The cell morphology and the mRNA expression levels of the marker genes of UC-MSCs was induced in different stages, dithizone staining of IPCs. **A** The cell morphology of UC-MSCs was induced in different stages, and Dithizone staining of IPCs, respectively. **B** The mRNA expression levels of the marker genes of UC-MSCs was induced in different stages, each sample in triplicates from three independent experiments; ** and **** indicate *p* < 0.05 and *p* < 0.001, and the difference is statistically significant

After in vitro induction of UC-MSCs, the cell morphology did not change significantly by adding β-mercaptoethanol combined with activin A in the first stage (S1 stage), compared with that of UC-MSCs without induction, which showed a typical long spindle shape and some cell death. In the second stage (S2 stage), the cells were further induced by removing β-mercaptoethanol over time. In the third stage (S3 stage), the cells were further shortened from the initial long fusiform shape to a flake shape, by reducing the serum concentration, and then adding bFGF, exendin-4, and NA. Finally, in the fourth stage (S4 stage), the cell morphology changed from a flake shape to an islet-like cluster shape by removing foetal bovine serum (FBS). In the fourth stage (IPCs), dithizone staining was scarlet, indicating that UC-MSCs were successfully differentiated into zinc-containing islet beta-like cells (Fig. 1A).

### mRNA expression levels and corresponding proteins during IPC differentiation

After collecting cells from each stage (S0, S1, S2, S3, and S4), RNA extraction and real-time fluorescence quantitative PCR and western blotting were performed. As shown in Fig. 1B, the marker genes of definitive endoderm Foxa2 and Sox17 from the first stage were increased significantly compared with those at other stages, the corresponding proteins were detected (Additional files 2, 8: Fig S2A, B), and the increase was statistically significant (*P* < 0.001). The pancreatic progenitor marker genes PDX1, Nkx6.1, and Ngn3 were increased significantly during the third stage compared with other stages, with a corresponding increase in PDX1 and Ngn3 protein (Additional files 2, 8: Fig S2 A, E, F). The increase was statistically significant (*P* < 0.05), indicating the successful induction of pancreatic precursor cells. Finally, the specific marker genes of islet beta-like cells Glut2, MAFA, and insulin were increased significantly in the fourth stage, with insulin protein also increased at fourth stage (Additional files 2, 8: Fig S2 A, D). This increase was also statistically significant (*P* < 0.001), while the α cell-specific marker gene glucagon and the Δ cell-specific marker gene somatostatin were not different significantly (*p* > 0.05) compared with control undifferentiated cells, as was glucagon protein (Additional files 2, 8: Fig S2A, C), indicating successful induction of islet beta-like cells.

### Immunofluorescence was used to detect the expression of key proteins during IPC identification.

**Fig. 2:**
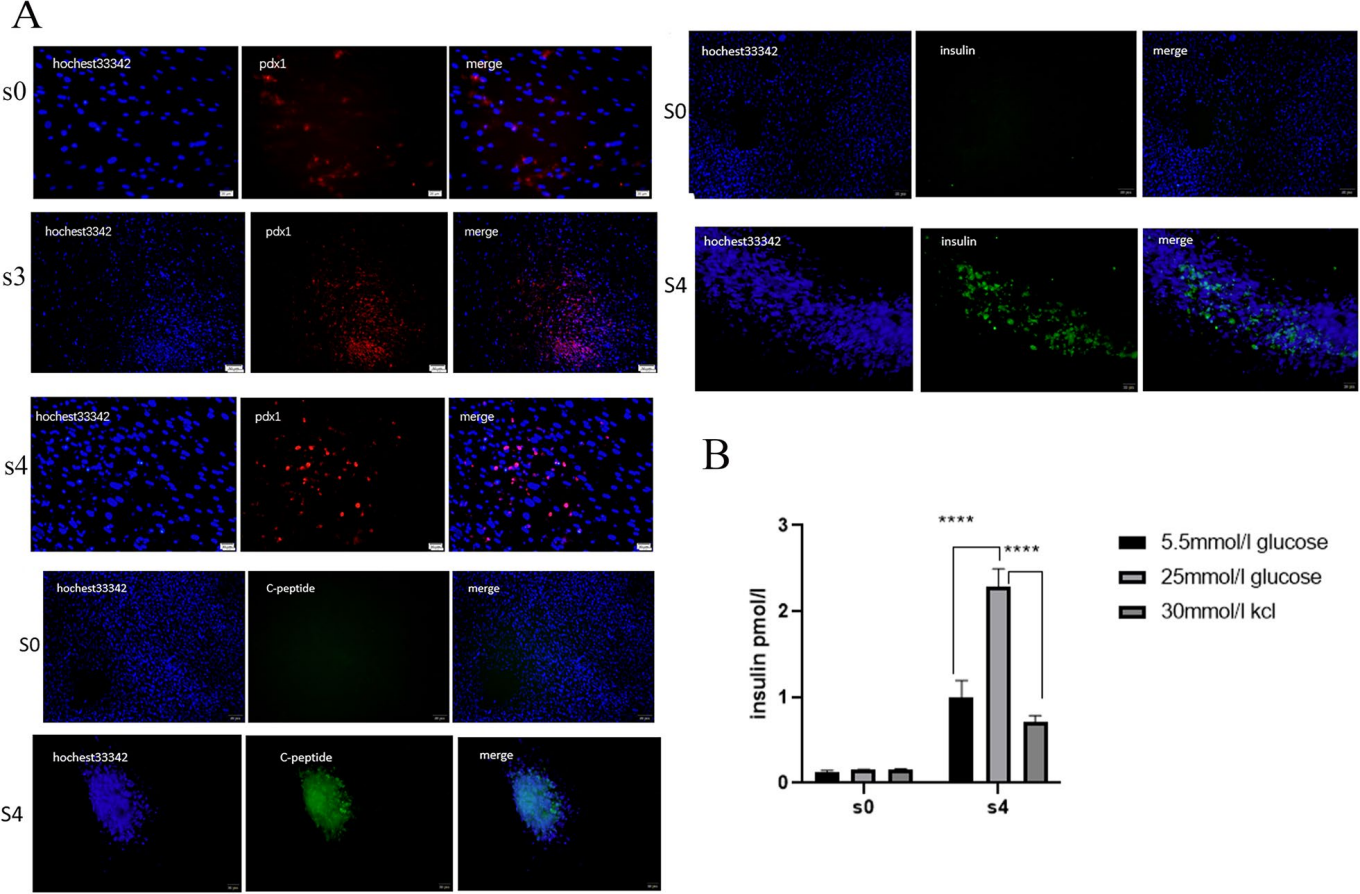
Immunofluorescence expression of key proteins at each stage and Glucose stimulation release experiment in vitro; each sample in triplicates from three independent experiments. **A** Immunofluorescence expression of key proteins at each stage; all images are magnified 100×; **B** glucose stimulation release experiment in vitro; ** indicates *p* < 0.05; the difference is statistically significant. All images have a resolution of 300dpi. All images are made using Photoshop. The intensity of Merge’s fluorescence signal was adjusted

After collecting cells from different stages (S0, S3, and S4), immunofluorescence experiments were performed. Figure 2A shows the changes in the expression of pdx1 protein in the S0, S3, and S4 stages and reveals the nuclear expression of pdx1. The figure shows that pdx1 was almost not expressed in the S0 stage, had the highest expression in the S3 stage and showed decreased expression in the S4 stage, which was consistent with the gene expression, indicating that it was successfully induced in pancreatic precursor cells. The diagram shows the changes in the expression of C-peptide protein in the S0 and S4 stages. The C-peptide was expressed in the cytoplasm. The picture shows that C-peptide was almost not expressed in the S0 stage, but was expressed in the S4 stage, which is consistent with the function of mature beta cells. The diagram shows the changes in the expression of insulin at the S0 and S4 stages. Insulin was expressed in the cytoplasm. The figure shows that insulin was almost not expressed in the S0 stage but was expressed in the S4 stage, which is consistent with the gene expression and the function of mature beta cells (Fig. 2A). In addition, we identified the expression of other key proteins at each stage. As shown in Additional file 3: Figure S3 and Additional file 4: Figure S4, immunofluorescence analysis demonstrated the nuclear expression of Foxa2, Sox17, and the cytoplasmic expression of GLUT2 and MAFA. Costaining for pdx1 and NKX6.1 at Day 15 (S3) indicated the presence of some double-positive beta-like cells, and pdx1 and insulin at Day 22 (S4) also revealed some double-positive beta-like cells.

### The function of IPCs was measured by a glucose stimulation test in vitro

Figure 2B shows the supernatant collected from UC-MSCs (S0) and IPCs (S4) at 22 days and incubated in low- and high-glucose solutions and 30 mM KCL solutions for 2 h. An insulin release assay was performed using a human insulin ELISA kit (Alpco, Salem, USA). The results showed that the amount of insulin released by the cells in the S0 stage in the 5.5 mM glucose solution, 25 mM glucose solution and 30 mM KCL solution did not differ (*P* < *0.05*). The amount of insulin released by the cells in the S4 stage in the 25 mM glucose solution was higher than that in the 5.5 mM glucose solution, and 30 mM KCL solution, and the difference was statistically significant (*P* < 0.001), which indicates that IPCs (S4) have functions similar to those of human pancreatic beta cells. All of the above results indicated that functional islet beta-like cells were successfully induced.

### Identification and global view of lncRNA profiles

**Fig. 3:**
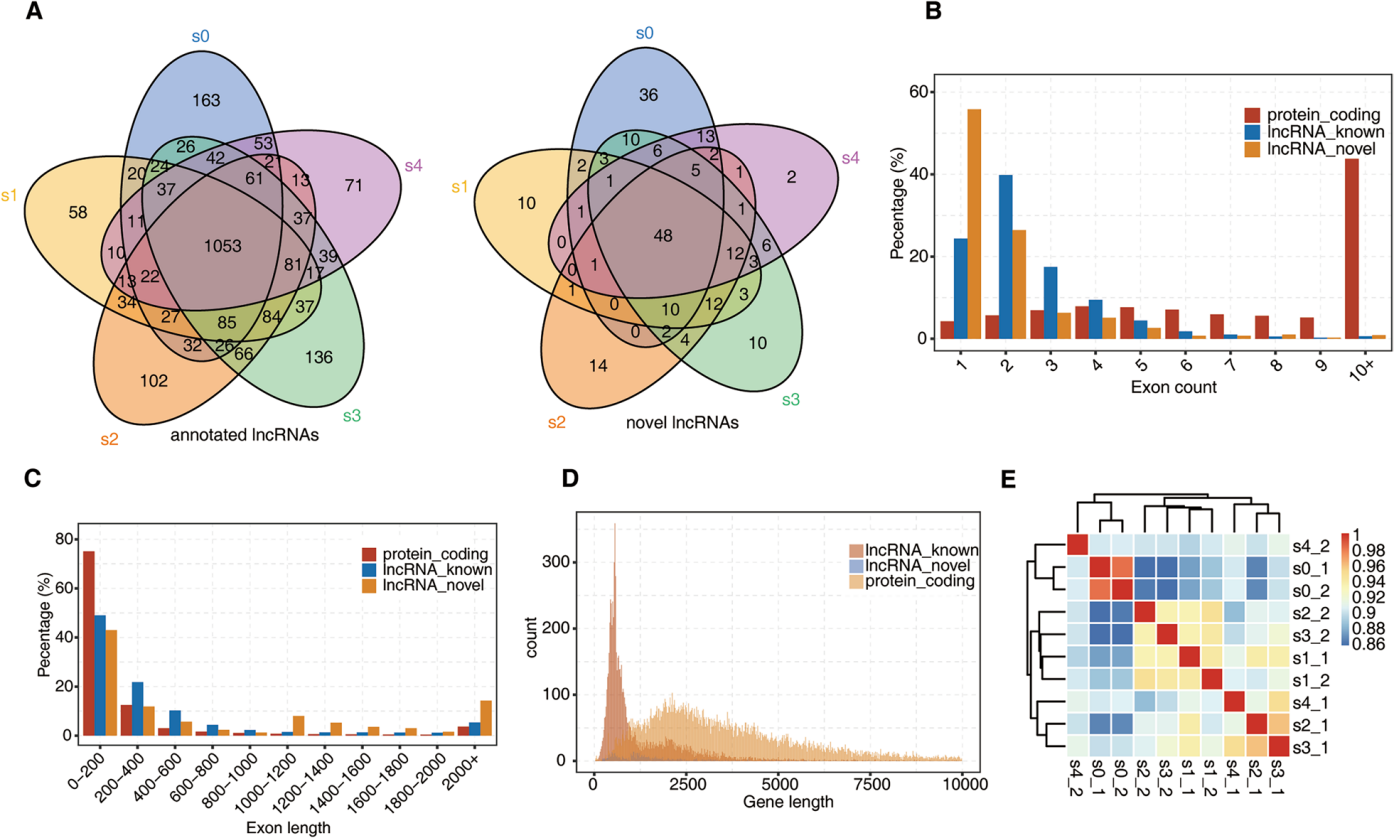
The profiling of differentially expressed lncRNAs during IPCs differentiation of UC-MSCs. **A** Venn diagram of detected known lncRNAs (left) and novel lncRNAs (right) in samples from five stages. At least two samples with RPKM >  = 0.2 was is considered to be detected in the group; **B** Distribution of exon count of known lncRNAs, novel lncRNAs, and protein-coding RNA; **C** Distribution of exon length of known lncRNAs, novel lncRNAs, and protein-coding RNA.; **D** Density of the length distribution of known lncRNAs, novel lncRNAs, and protein-coding RNA. The length density distribution was generated by density function in R; **E** Heatmap clustering analysis of sample correlation based on the normalized mapped reads on each lncRNAs

We generated cDNA libraries of polyadenylated RNA extracted from ten samples and RNA-seq data sets at a sequencing depth of 8.6 million reads per sample, and the average number of reads entering the mapping process across all analysed samples was 84.9 million. The average percentage of uniquely mapped reads was 92.81%, and the average percentage of reads mapped to multiple loci was 3.09% (Additional file 9: Table S1). Some results of the quality control of the RNA-seq data sets are shown (Additional file 5: Fig S5A-C). We aligned the filtered reads to the reference sequence (human GRch38 genome) by HISAT2, predicted lncRNAs and calculated their expression levels using Cufflinks. The number of annotated lncRNAs and novel lncRNAs of in each of the five stages was profiled. Among them, 1053 annotated lncRNAs and 48 novel lncRNAs were continuously expressed across all five stages (Fig. 3A). LncRNAs obtained by screening included lincRNAs (30.4%), antisense lncRNAs (15.8%), and sense-overlapping lncRNAs (53.8%) (Additional file 5: Fig. S5D). Most lncRNAs contained approximately 1 or 2 exons and no more than 10 exons; however, most protein-coding transcripts contained more than 10 exons (Fig. 3B). In addition, a length of 0–200 bp represented the dominant portion of the exon length of the lncRNAs and protein-coding transcripts (Fig. 3C). The length of the dominant portion of lncRNAs was around 1000 bp, while the length of protein-coding transcripts was nearly 2500 bp (Fig. 3D). Overall, lncRNAs have fewer exons than protein-coding transcripts, and the lengths of these lncRNAs are generally shorter than those of mRNAs. To understand the quality control of the experimental data, a Pearson correlation analysis was performed for all pairs of RNA-seq samples, and it demonstrated that the correlation coefficient between each repeated sample reached 0.8, indicating that the overall quality of the data was good (Fig. 3E, Additional file 6: Fig S6. A).

### Differentially expressed lncRNAs and functional enrichment of target genes

**Fig. 4:**
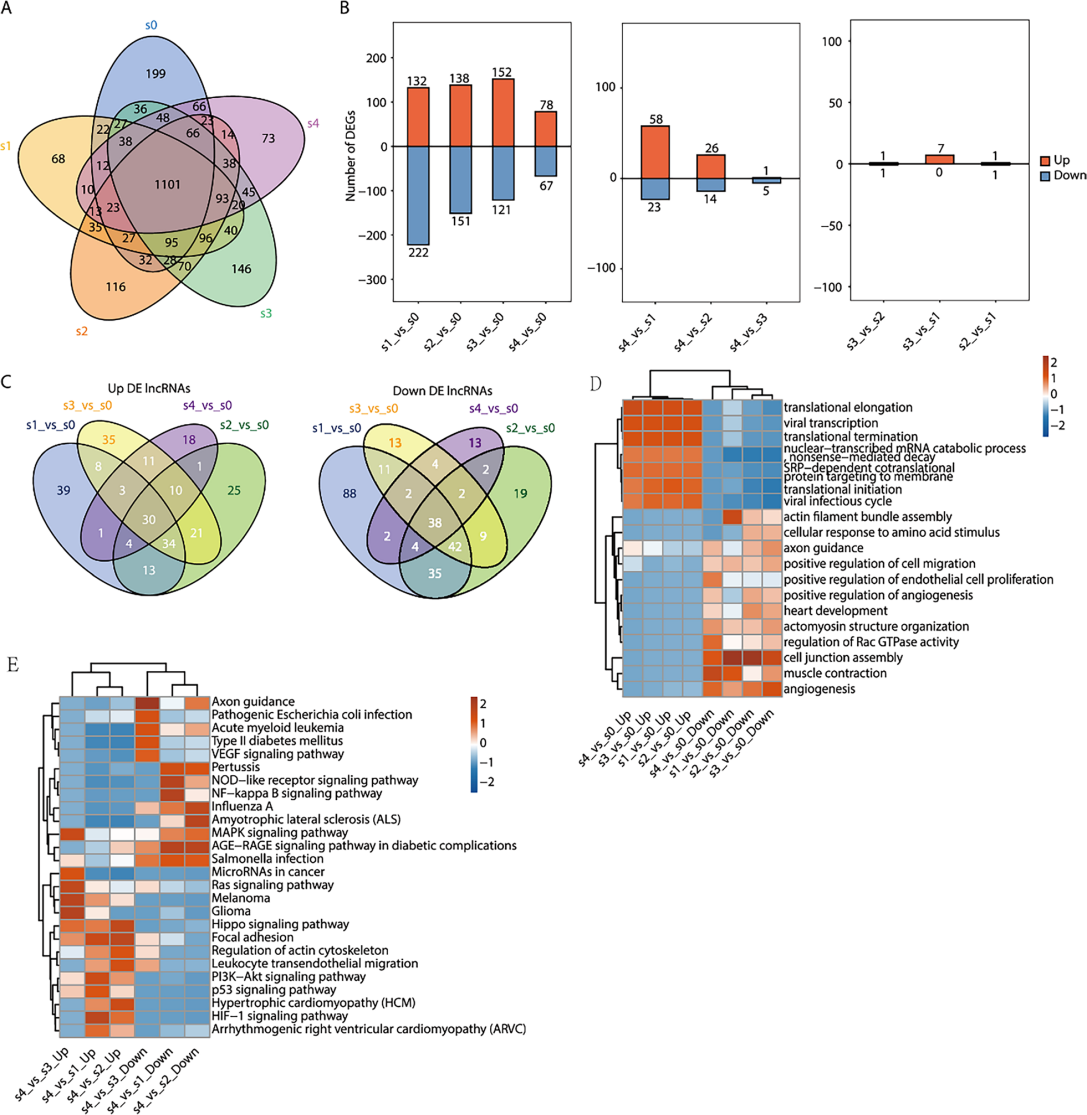
The expression of LncRNAs at each stage and functional enrichment of target genes of differentially expressed lncRNAs. **A** Venn diagrams of LncRNAs detected in samples of 5 stages, the p value or corrected p value (padj) was used to determine the level of significance for multiple samples; **B** bar plots of differential LncRNAs at different stages; **C** the Venn diagram showed the overlap of differentially up-regulated or down-regulated LncRNAs (S0 stage was compared with S1, S2, S3, and S4 stages, respectively); **D** S0 stage was compared with S1, S2, S3, and S4 stages, respectively, and GO(molecular process) enrichment analysis of the mRNA of differentially up-regulated or down-regulated LncRNAs, the colour scale shows the significance of these terms scaled by column (− log10 corrected *p* value); **E** S4 stage was compared with S1,S2,and S3 stages, respectively, and KEGG enrichment analysis of the mRNA of differentially up-regulated or down-regulated LncRNAs, the colour scale shows the significance of these terms scaled by column(− log10 corrected *p* value)

To identify the differentially expressed lncRNAs during IPC differentiation of UC-MSCs, the number of lncRNAs was detected in each five stages. A total of 2615 lncRNAs with differential expression were identified, of which 1101 lncRNAs were continuously expressed across all five stages (Fig. 4A). To further explore changes in the dynamic expression of differentially expressed lncRNAs across the five stages during induction, the S0 stage was compared with the S1, S2, S3, and S4 stages, the S4 stage was compared with the S1, S2, and S3 stages, the S3 stage was compared with the S2 and S1 stages, and the S2 stage was compared with the S1 stage. Up- or down-regulated lncRNAs were identified, which revealed that the number of differentially expressed lncRNAs among them decreased successively. Moreover, mRNAs levels followed the same expression trend (Fig. 4B, Additional file 6: Fig. S6B). The overlap of differentially expressed lncRNAs is shown in the S1, S2, S3, and S4 stages compared with the S0 stage. Among them, 30 lncRNAs were continuously up-regulated, and 38 lncRNAs were continuously down-regulated across the five stages. The overlap of differentially expressed mRNAs is shown (Fig. 4C, Additional file 6: Fig. S6C).

To identify the function of differentially expressed lncRNAs that controlled IPC differentiation of UC-MSCs, we performed Gene Ontology (GO) analysis and pathway analysis. Since lncRNAs do not encode proteins, their regulatory effect can only be exerted by regulating the genes coexpressed with them. First, GO analysis was performed to identify enrichment in the significant functions of the genes coexpressed with differentially expressed lncRNAs, which were obtained during IPC differentiation of UC-MSCs. We obtained the top 20 enriched GO functions according to the *p* value and FDR (*p* < 0.05, FDR < 0.05) in the S1, S2, S3, and S4 stages compared with the S0 stage. Coexpressed genes of highly expressed lncRNAs in the S1, S2, S3, and S4 stages were mainly concentrated in protein translation-related pathways. In contrast, the coexpressed genes of lncRNAs with low expression were mainly enriched in the cell adhesion and cell connection pathways (Fig. 4D). The top 20 enriched GO functions of differentially expressed mRNAs are shown (Additional file 6: Fig. S6D).

We then identified enrichment in the significantly changed pathways that mediated the functions of the genes coexpressed with differentially expressed lncRNAs based on the KEGG database. The top 5 significant pathways in stage S4 compared with stages S1, S2, and S3 were related to the insulin signal transduction pathway of beta cells, and they may play key roles in the function of pancreatic beta cells. Among them, up-regulated lncRNAs were mainly enriched in the NF-KB signalling pathway and MAPK signalling pathway, and down-regulated lncRNAs were mainly enriched in the HIPPO signalling pathway, PI3K–Akt signalling pathway, p53 signalling pathway, and other signalling pathways. In summary, the KEGG analysis revealed that differentially expressed lncRNAs were related to the insulin signal transduction pathway of beta cells (Fig. 4E).

### The expression patterns of lncRNAs in the S1, S2, S3, and S4 stages were demonstrated by K-means analysis

**Fig. 5:**
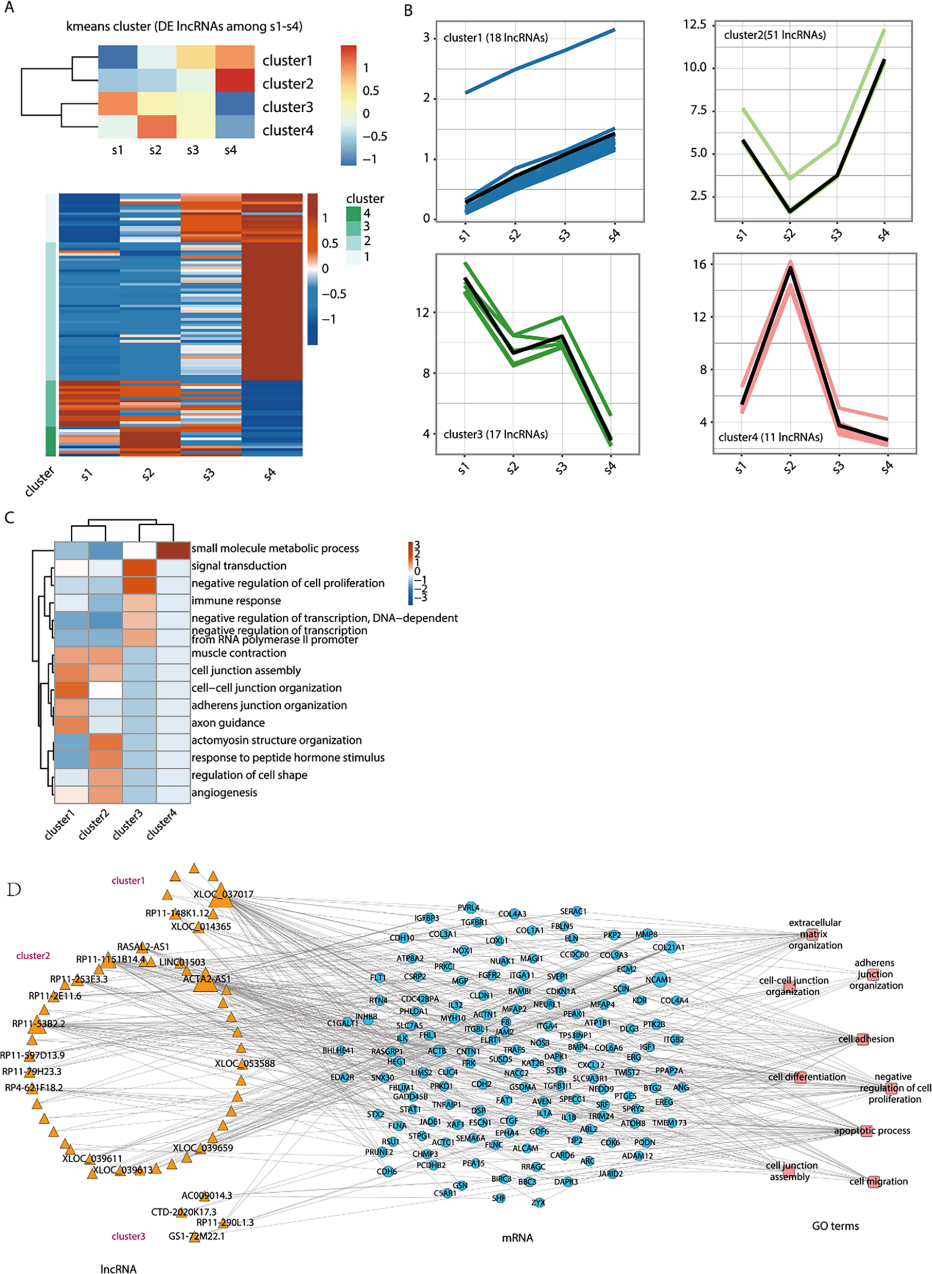
The expression pattern of LncRNAs in S1 stage to S4 stage was demonstrated by K-means analysis. **A** Differential LncRNAs in S1 stage to S4 stage were clustered by K-means; **B** LncRNAs expression profiles generated by K-means clustering;** C** the top 5 GO terms (molecular processes) of coexpressed mRNAs with 4 clusters of differential LncRNAs generated by K-means clustering, and the colour scale shows the significance of these terms scaled by column (− log10 corrected *p* value). **D** Coexpression network of co-regulated LncRNAs in gene clusters 1 to 3 and coexpression mRNAs participating in 9 GO terms

To characterize the dynamic changes in lncRNA and mRNA expression, we clustered all their expression patterns (97 lncRNAs and 769 mRNAs) by K-means analysis. We identified 4 lncRNA and mRNA clusters. The upper part of the module shows four clusters identified by K-means, and the lower part shows the lncRNA and mRNA expression profiles of four clusters (Fig. 5A, Additional file 7: Fig. S7A). The lncRNA expression patterns in the four clusters are displayed, among which the trend in expression levels of lncRNAs in Clusters 1 and 3 was consistent with that of the mRNAs (Fig. 5B, Additional file 7: Fig. S7B). The top 5 signalling pathways of genes coexpressed with lncRNAs in the four different clusters are displayed (Fig. 5C). Moreover, we found that mRNA Cluster 1 mainly showed enrichment in the cell proliferation and cell differentiation pathways, indicating that cell differentiation tended was active, while Cluster 3 was mainly concentrated in the negative regulation of cell proliferation and the cell adhesion pathway. Thus, with the development of cell differentiation, the ability to inhibit cell proliferation and the connection between cells became weaker. Namely, cell differentiation became increasingly active, which was in line with the process of cell differentiation according to the functional enrichment analysis of the four mRNA clusters (Additional file 7: Fig. S7C). The expression levels of the P53, FIGF, JARID2, and PROX1 genes, which are involved in the development and function of pancreatic beta cells in Clusters 1 and 3, were determined (Additional file 7: Fig. S7D). To explore the functions of lncRNAs, the network interactions of lncRNAs and their coexpressed mRNAs in Clusters 1–3 and the enrichment pathways related to cell differentiation are shown (Fig. 5D).

### Screening of pancreatic development-related lncRNAs and their coexpressed mRNAs

**Fig. 6:**
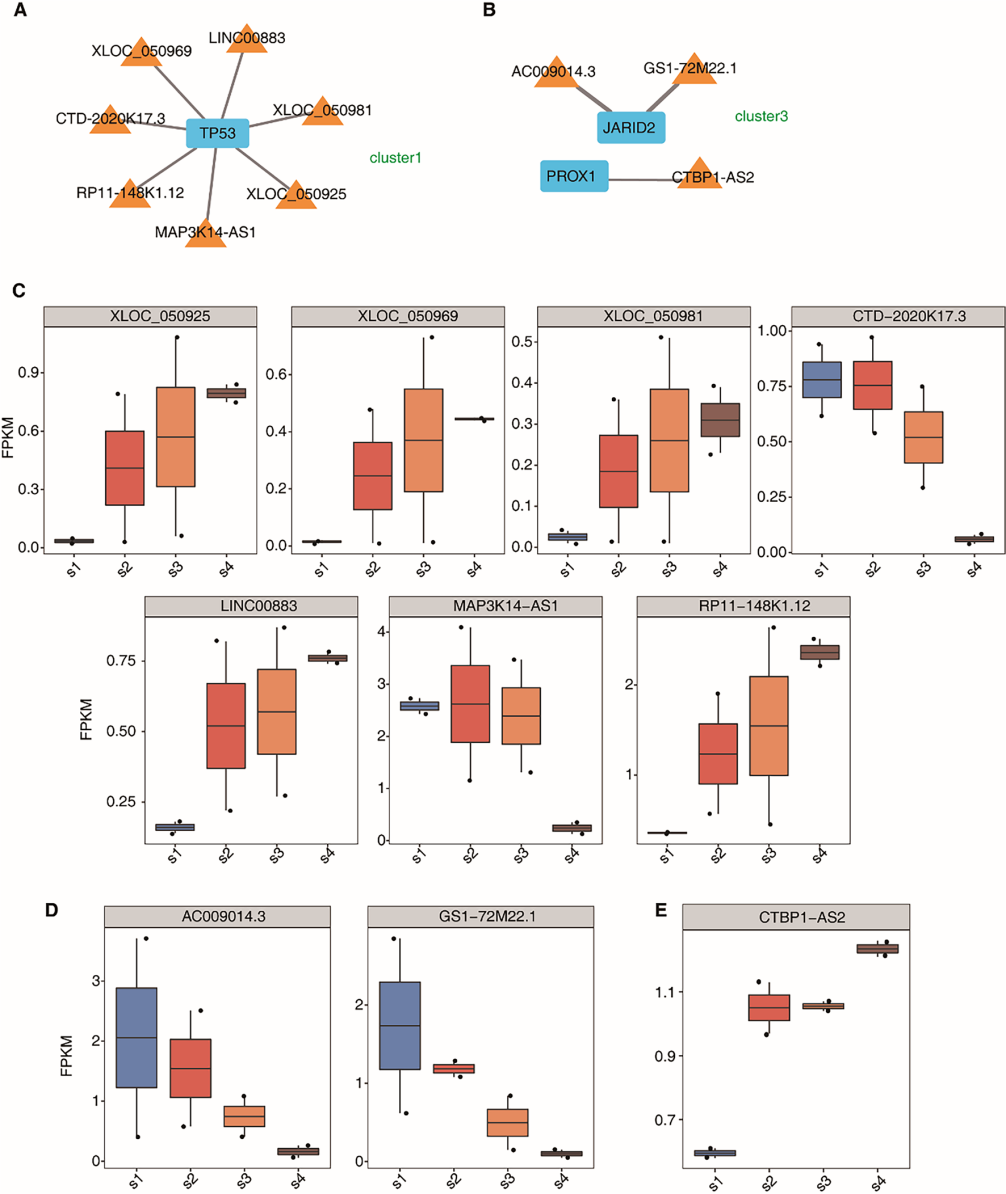
The expression trend of coexpressed LncRNAs in differentiation. **A** The panel showed the coexpression network of coexpressed LncRNAs with TP53 in cluster1; **B** the panel showed the coexpression network of coexpressed LncRNAs with JARID2 and RPOX1 in cluster3; **C** boxplot of expression levels coexpressed LncRNAs with TP53 at various stages; **D** boxplot of expression levels coexpressed LncRNAs with JARID2 at various stages; **E** boxplot of expression levels of coexpressed LncRNAs with RPOX1 at various stages

**Fig. 7:**
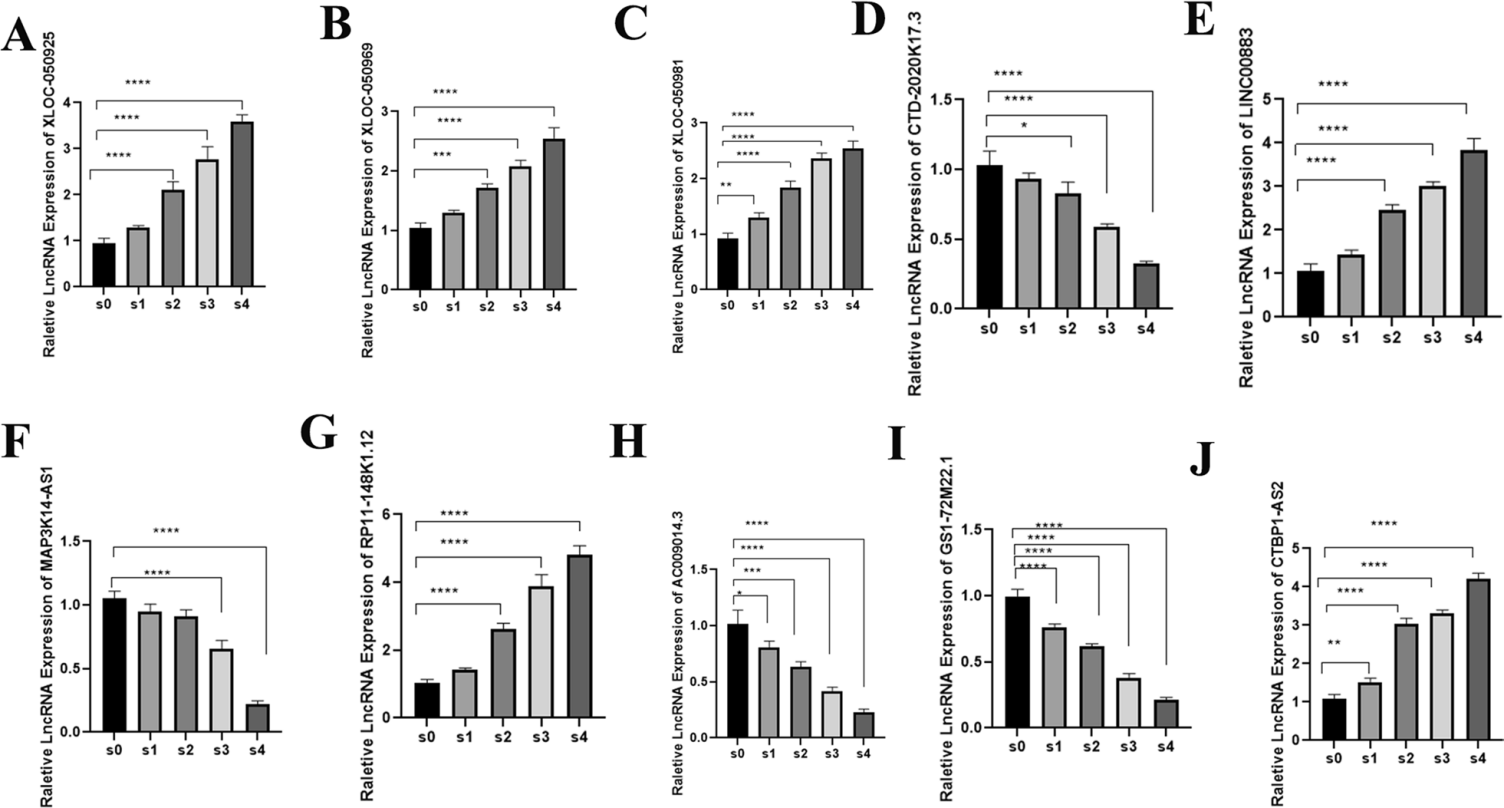
Validation of ten differentially expressed lncRNAs. **represents *P* < 0.05, ****represents *P* < 0.001, and the difference is statistically significant

Since the focus of our study was to investigate lncRNAs associated with IPC differentiation, we screened the mRNAs JARID2, PROX1, and p53, which are related to the development and function of the pancreas in the functional enrichment pathway. Cervanets et al. found that JARID2 plays a role in the late differentiation of embryonic pancreatic beta cells. Pethe et al. found that the expression levels of JARID2 decreased gradually during directed differentiation and spontaneous differentiation of the pancreatic system. Studies have also found that PROX1 is a specific marker of the endoderm in early pancreatic development. Other studies have found that PROX1 is highly expressed in pancreatic endocrine progenitor cells, but is lacking in mature beta cells. PROX1 activity is essential for the formation of endocrine progenitor cells and the differentiation of α cells [35–39]. Paul et al. found that beta cells overexpressing Prox1 rapidly inactivate *MafA* after birth, and downregulating Prox1 is a prerequisite to expand the β-cell mass after birth and for proper maturation of this lineage [40]. The activity of *MafA* plays an important role in beta cell maturation because it governs the glucose-responsive transcription of insulin and key components of the glucose-stimulated insulin secretion mechanism [41, 42]. Kung et al. demonstrated that p53 regulates insulin secretion and pancreatic beta cell survival through multiple signalling pathways [43]. The coexpression of the lncRNAs:AC009014.3 and Gs1-72m21.1 with JARID2 and lncRNA CTBP1-AS2 with PROX1 was identified through coexpression analysis (Fig. 6B, D, E). Omidvar et al. found that lncRNA CTBP1-AS2 expression levels are associated with T2D susceptibility [44], which means that lncRNA CTBP1-AS2 may regulate pancreatic maturation by regulating PROX1 mRNA. These results indicate that the lncRNAs AC009014.3, Gs1-72m21.1 and CTBP1-AS2 may be involved in the development of pancreatic beta cells. The lncRNAs XLOC_050969, LINC00883, XLOC_050981, XLOC-050925, MAP3K14-AS1, RP11148K1.12, and CTD2020K17.3 were coexpressed with p53 and may be involved in regulating insulin secretion by pancreatic beta cells. The expression trend of corresponding lncRNAs at all stages of induction was also analysed and verified by qPCR (Fig. 6AC). The qPCR results were consistent with the RNA-seq data (Fig. 7A–J).

## Discussion

Long noncoding RNAs (lncRNAs) are the largest component of the mammalian noncoding transcriptome, with a length greater than 200 nt [45]. According to the genomic position of lncRNAs relative to adjacent or overlapping protein-coding genes, they can be divided into sense, antisense, intron, intergenic or enhancer lncRNAs that mediate short- and long-range interactions between transcriptional enhancers and other regulatory elements in the genome [46]. lncRNAs can control the expression of cis or trans genes by directly interacting with transcription factors or recruiting chromatin modification complexes [47–49]. The expression levels of lncRNAs are generally lower than those of mRNAs, although a large proportion of the identified lncRNAs have high islet specificity, with a dynamic modulated pattern observed in differentiated beta-like cells in vitro, and most detected lncRNAs have increased specificity in functional endocrine differentiated cells [50]. Akerman et al. found that the lncRNAs HI-LNC-12, 15, 30, 78, 80, and 71 significantly affected the steady-state transcription levels of transcription factors of beta cells such as PDX1 and HNF1A. HI-LNC-12, HI-LNC-78, and HI-LNC-71 knockdown (KD) inhibited insulin secretion and reduced the insulin content in Endoc cells stimulated by glucose, and the expression levels of many lncRNAs were highly correlated with transcription factors (GLIS3, HNF1A, NKX2.2, PDX1, and MAFB) in beta cells [51]. Therefore, lncRNAs play important roles in the development of pancreatic beta cells and perform regulatory functions in pancreatic beta cells.

In this study, differentially expressed lncRNAs and mRNAs were obtained during the differentiation of stem cells into insulin-producing cells. GO analysis of these differentially expressed lncRNAs showed that the up-regulated genes were mainly functionally enriched in translation-related pathways, such as the initiation, extension, and termination of translation, and down-regulated genes were mainly concentrated in cell connection and adhesion and extracellular matrix tissue pathways. Differentially expressed lncRNAs had more pathway enrichment in the S4 stage compared with the S1, S2, and S3 stages based on KEGG analysis. Many target genes were involved in the *NF-KB* signalling pathway, MAPK signalling pathway, HIPPO signalling pathway, PI3K–Akt signalling pathway, and *p53* signalling pathway. In a study of beta cells treated with free fatty acids that mimic pancreatic dysfunction and apoptosis induced by gut obesity, activation of *p53* led to the induction of miR34a, a microRNA that sensitizes beta cells to apoptosis and inhibits the insulin exocytosis pathway, thus leading to impaired insulin secretion [52]. It has been shown that in beta cells, elevated glucose levels lead to elevated *Ca*^*2*+^ levels and enhanced *NF-KB* activity, which promotes insulin release. Overexpression of IKBa inhibits intracellular *NF-KB* activity and leads to impaired glucose-stimulated insulin secretion [53]. Studies have shown that *PI3K-Akt* is one of the major signalling pathways that maintains the survival and replication of beta cells and the expression and secretion of insulin genes [53–55]. The above discussion indicates that these pathways are related to the insulin secretion of islet beta cells.

LncRNAs can promote the differentiation of stem cells by interacting with miRNAs. Huang et al. found that lncRNA Gm10451 could regulate the histone H3K4 methyltransferase complex PTIP to facilitate insulin^+^/Nkx6.1^+^ beta-like cell differentiation by targeting miR-338-3p as a competing endogenous RNA (ceRNA), and Gm10451 loss in beta-like cells prevented the expression of mature beta cell markers, such as insulin, Nkx6.1, and MafA, after transplantation into streptozotocin (STZ)-mice [56]. Zou et al. demonstrated that lncRNA-ROR effectively maintains Sox2 gene expression through competitive binding to miR-145, thereby improving the efficiency of human amniotic epithelial stem cell (HuAEC) differentiation into beta islet-like cells [57].

In this research, we found that lncRNA CTBP1-AS2 is coexpressed with PROX1, PROX1 regulates pancreatic beta cell maturation by affecting MafA, and lncRNA CTBP1-AS2 is associated with T2DM. Therefore, lncRNA CTBP1-AS2 may promote beta cell maturation by regulating PROX1 and then MafA, which may be the direction of our future research. This also provides a new idea for improving the efficiency of the differentiation of stem cells into insulin-producing cells in the future.

## Conclusion

In conclusion, HUC-MSCs combined with small molecule compounds were successfully induced to differentiate into IPCs. Differentially expressed lncRNAs may regulate the insulin secretion of pancreatic beta cells by regulating multiple signalling pathways. The lncRNAs AC009014.3, Gs1-72m21.1 and CTBP1-AS2 may be involved in the development of pancreatic beta cells, and the lncRNAs**:** XLOC_050969, LINC00883, XLOC_050981, XLOC_050925, MAP3K14-AS1, RP11-148K1.12, and CTD2020K17.3 may be involved in regulating the insulin secretion of pancreatic beta cells. However, the specific mechanisms underlying the involvement of these lncRNAs in the regulation of pancreatic development and insulin secretion of pancreatic beta cells need to be further explored, and the lncRNA CTBP1-AS2 will be our focus. These findings expand the lncRNA database relating to stem cell differentiation of IPCs and can also serve as a reliable foundation for further research.

### Supplementary Information

**Additional file 1: Figure S1. **The cell morphology and identification of UC-MSCs.** A** The cell morphology of UC-MSCs crawling out of the tissue on the 7th day, and the cell morphology of the first passage of UC-MSCs on the 9th day; all images are magnified 40x;** B** the phenotype of UC-MSCs in vitro was identified by flow cytometry: the expression of CD34, HLA-DR, CD45, CD14, CD73, CD90, CD105, and CD44, respectively;** C** The identification of UC-MSCs differentiation ability: UC-MSCs by alizarin red staining, Alcian blue staining, and Oil red O staining, respectively, all images are magnified 100x.

**Additional file 2:** **Figure S2.** Western blotting assays were conducted to evaluate the expression of key protein (**A**-**F**). Supplementary file of Fig. S2 shows the original blots of each protein in Fig. S2A. **** indicates *p* < 0.001, and the difference is statistically significant.

**Additional file 3: Figure S3.** Immunofluorescence expression of key proteins at S1 and S3 stage. All images aremagnified 100x. All images have a resolution of 300dpi. All images are made using Photoshop. The intensity of Merge's fluorescence signal was adjusted.

**Additional file 4: Figure S4.** Immunofluorescence expression of key proteins at S4 stage. All images are magnified 100x. All images have a resolution of 300dpi. All images are made using Photoshop. The intensity of Merge’s fluorescence signal was adjusted.

**Additional file 5: Figure S5. **The quality control of RNA-seq data sets and the classification of lncRNAs (take one of these samples for example).** A** sequencing data filtering;** B** sequencing error rate distribution;** C** GC content distribution;** D** genomic regional distribution.

**Additional file 6: Figure S6. **The expression of mRNAs at each stage and functional enrichment of differentially expressed mRNAs.** A** Heatmap clustering analysis of sample correlation based on the normalized mapped reads on each mRNAs; **B** bar plots of differential mRNAs at different stages; **C** the Venn diagram showed the overlap of differentially up-regulated or down-regulated mRNAs (S0 stage was compared with S1, S2, S3, and S4 stages, respectively); **D** S0 stage was compared with S1, S2, S3, and S4 stages, respectively, and GO (molecular process) enrichment analysis of differentially up-regulated or down-regulated mRNAs; the colour scale shows the significance of these terms scaled by column (-log10 corrected p value).

**Additional file 7:** **Figure S7. **The expression pattern diagram of mRNAs in S1 stage to S4 stage was displayed by K-means analysis.** A** Differential mRNAs in S1 stage to S4 stage were clustered by K-means;** B** Gene expression profiles generated by K-means clustering; **C** the four clusters of differential mRNAs generated by K-means clustering showed the top five most enriched GO terms (molecular processes); the colour scale shows the significance of these terms by column (-log10 corrected p value); **D** the expression of four mRNAs at each stage.

**Additional file 8:** Original image of the protein in Fig. S2A.

**Additional file 9: Table S1**. The quality control of RNA-seq data sets.

**Additional file 10: Table S2**. Relevant antibody information for the Western blot.

## Data Availability

The data sets used and/or analysed during the current study have been deposited in the NCBI under accession numbers (GSE247570) (Home—GEO—NCBI (nih.gov)).

## Abbreviations

ADSC: Adipose tissue-derived stromal cells
BMSC: Bone marrow stromal cells
FPKM: Fragments per kilobase of exon per million fragments mapped
GO: Gene ONTOLOGY
HUC-MSCs: Human umbilical cord mesenchymal stem cells
IPCs: Insulin-producing cells
LncRNA: Long noncoding RNA
