# Supplementary figures and images for: Dysregulated lncRNAs regulate human umbilical cord mesenchymal stem cell differentiation into insulin-producing cells by forming a regulatory network with mRNAs
Source: Stem Cell Res Ther. 2024 Jan 25;15:22. doi: 10.1186/s13287-023-03572-5 (PMC10809572; doi:10.1186/s13287-023-03572-5)

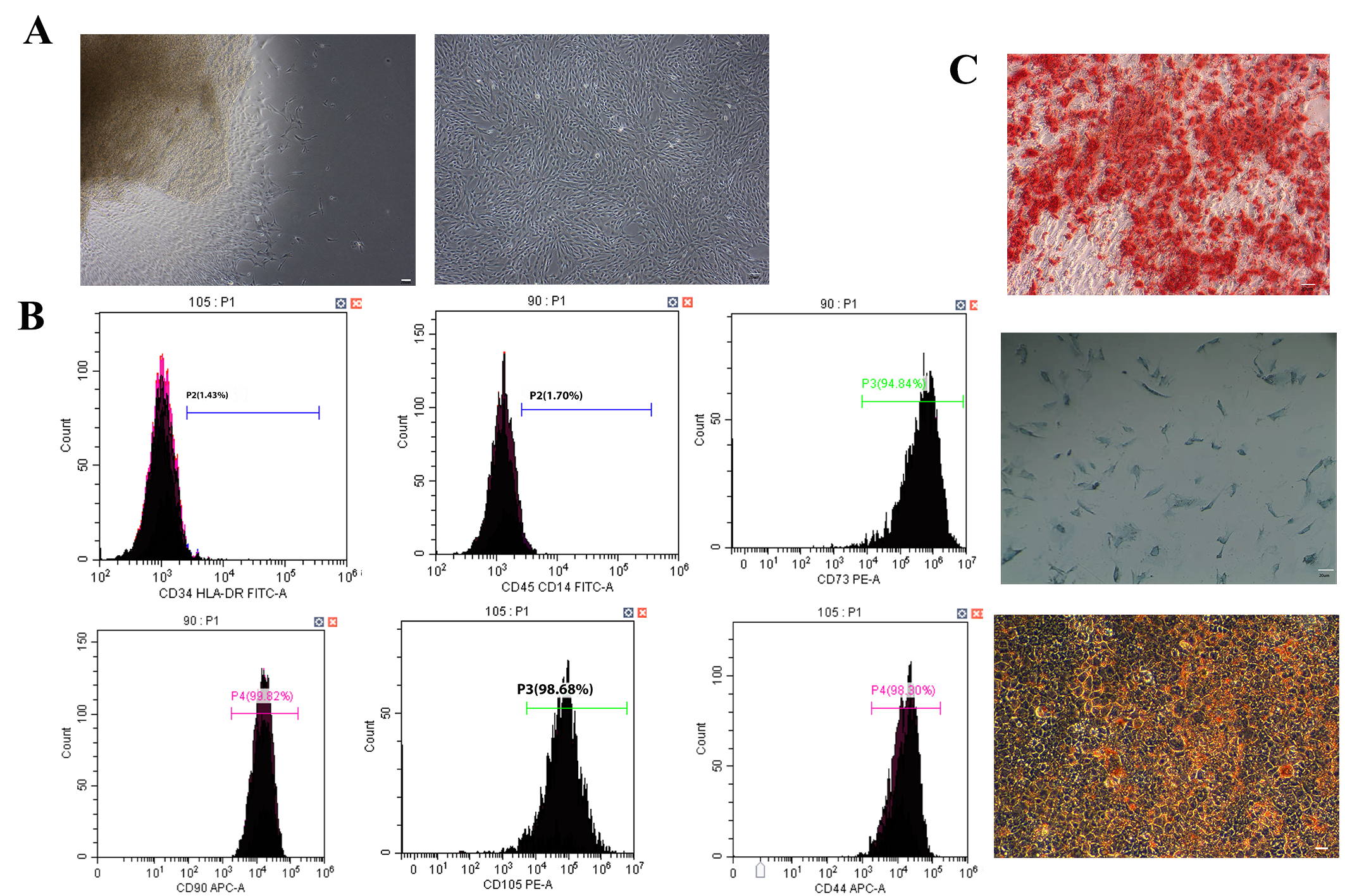

Supplement: Supplementary file 1 — Additional file 1: Figure S1. The cell morphology and identification of UC-MSCs. A The cell morphology of UC-MSCs crawling out of the tissue on the 7th day, and the cell morphology of the first passage of UC-MSCs on the 9th day; all images are magnified 40x; B the phenotype of UC-MSCs in vitro was identified by flow cytometry: the expression of CD34, HLA-DR, CD45, CD14, CD73, CD90, CD105, and CD44, respectively; C The identification of UC-MSCs differentiation ability: UC-MSCs by alizarin red staining, Alcian blue staining, and Oil red O staining, respectively, all images are magnified 100x. [file 13287_2023_3572_MOESM1_ESM.tif]

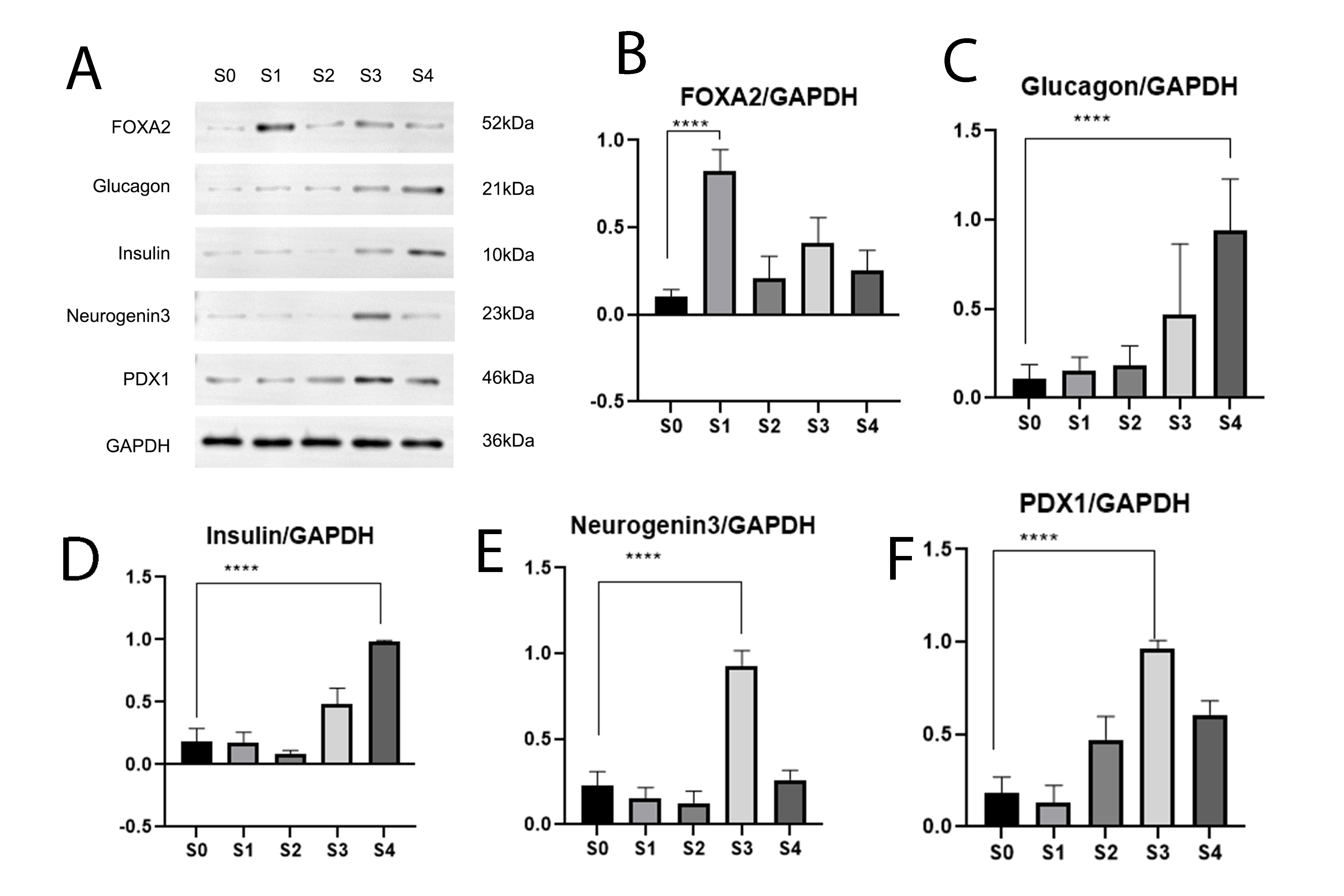

Supplement: Supplementary file 2 — Additional file 2: Figure S2. Western blotting assays were conducted to evaluate the expression of key protein (A-F). Supplementary file of Fig. S2 shows the original blots of each protein in Fig. S2A. **** indicates p < 0.001, and the difference is statistically significant. [file 13287_2023_3572_MOESM2_ESM.tif]

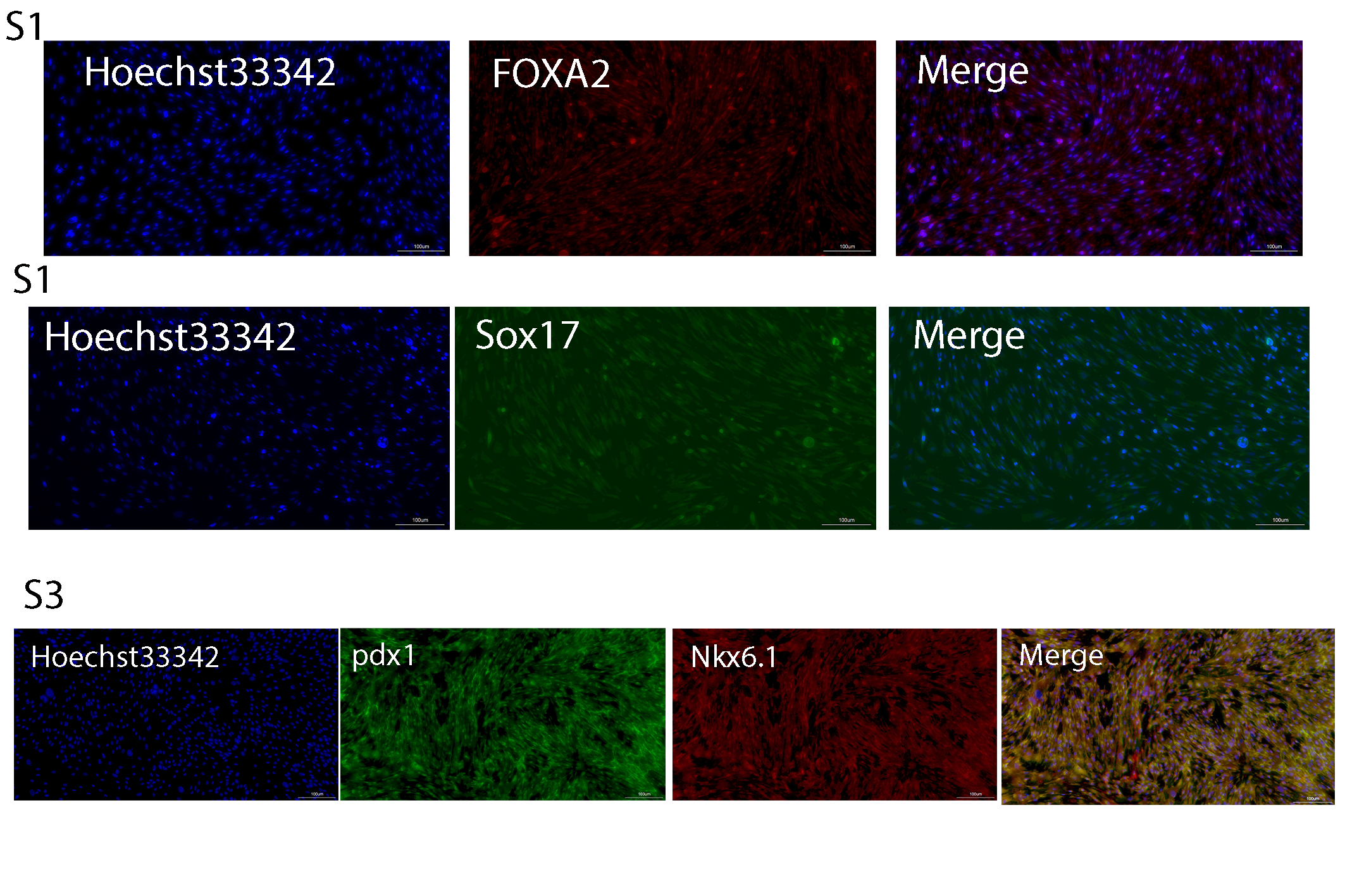

Supplement: Supplementary file 3 — Additional file 3: Figure S3. Immunofluorescence expression of key proteins at S1 and S3 stage. All images aremagnified 100x. All images have a resolution of 300dpi. All images are made using Photoshop. The intensity of Merge's fluorescence signal was adjusted. [file 13287_2023_3572_MOESM3_ESM.tif]

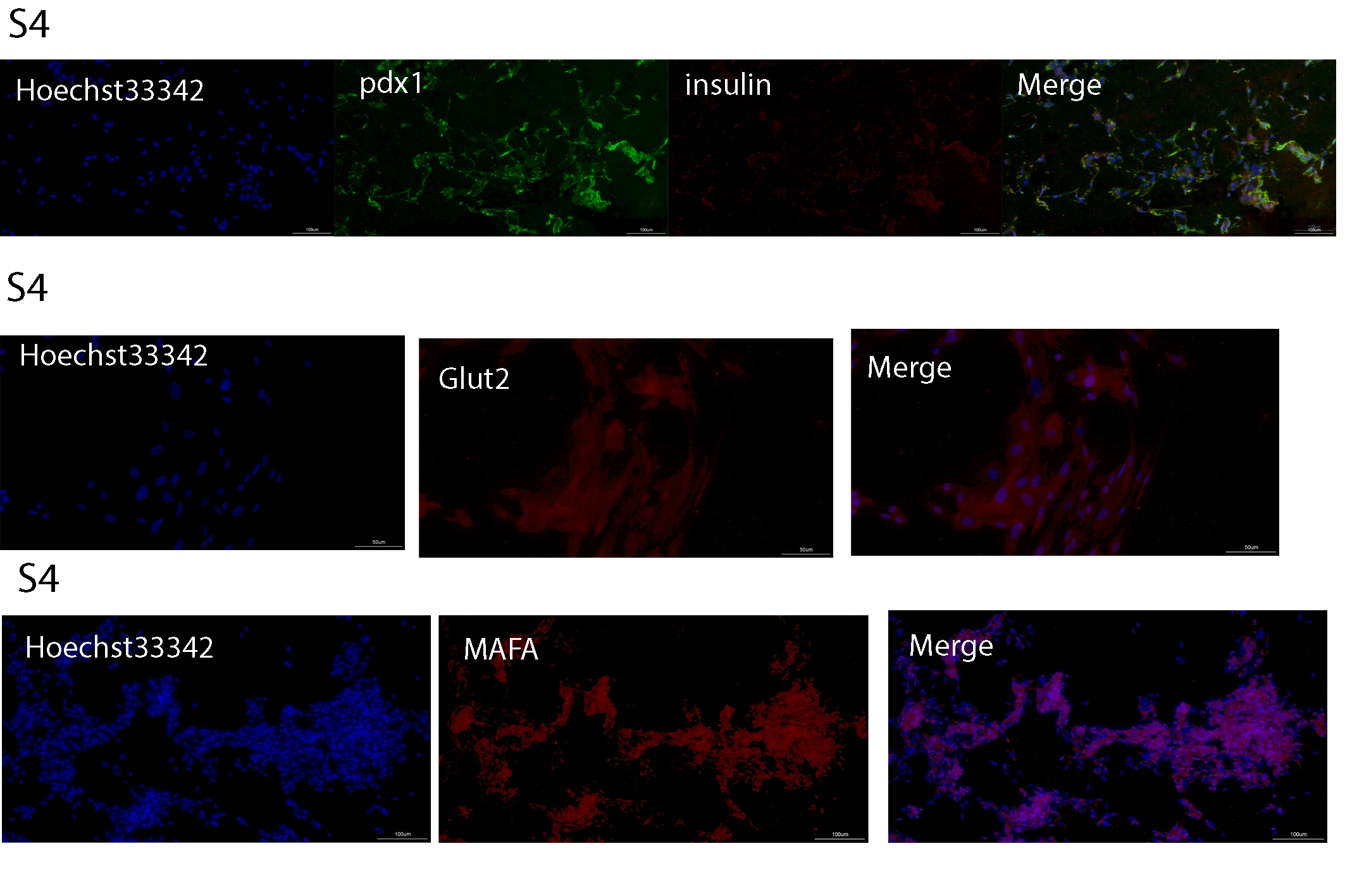

Supplement: Supplementary file 4 — Additional file 4: Figure S4. Immunofluorescence expression of key proteins at S4 stage. All images are magnified 100x. All images have a resolution of 300dpi. All images are made using Photoshop. The intensity of Merge’s fluorescence signal was adjusted. [file 13287_2023_3572_MOESM4_ESM.tif]

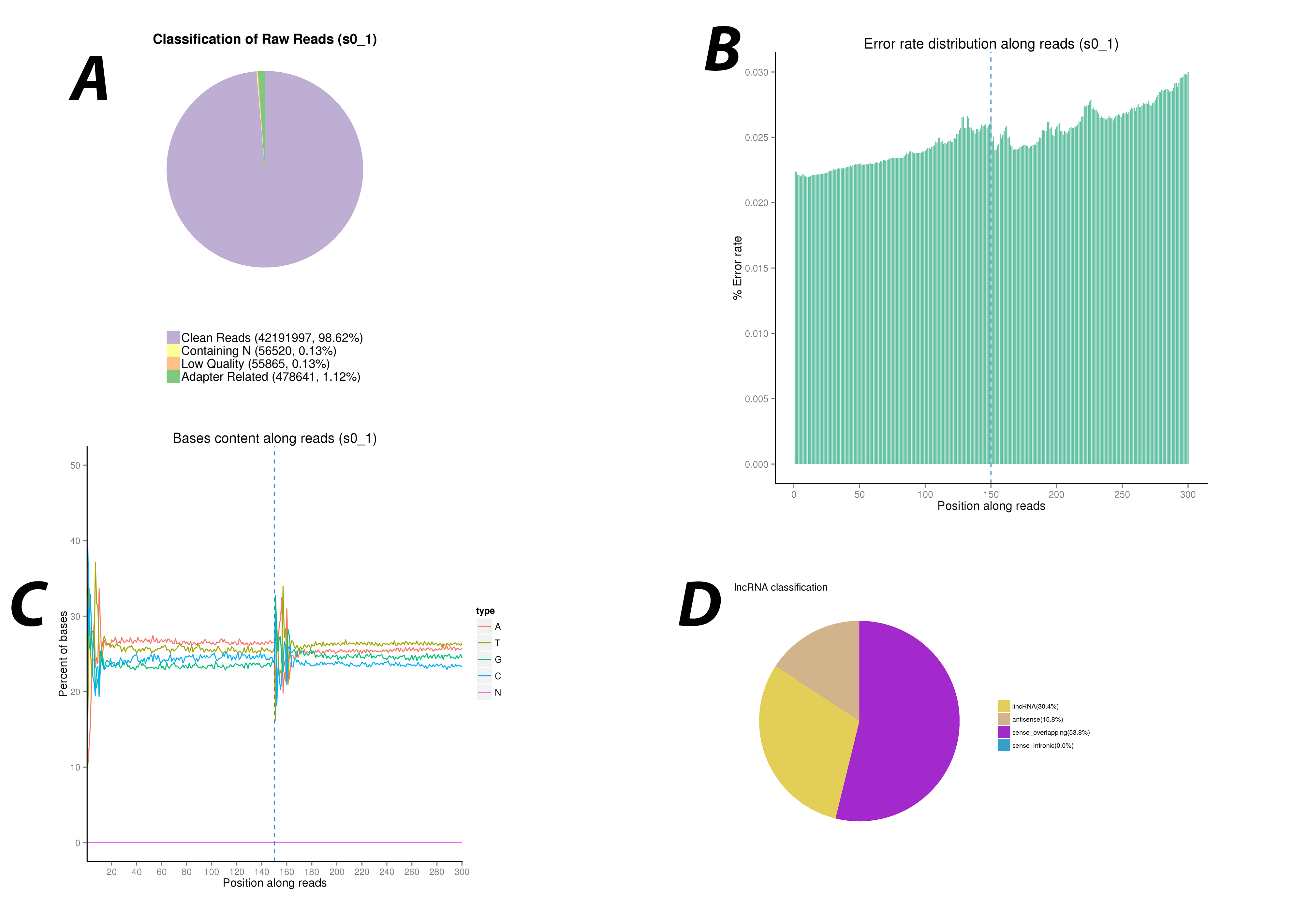

Supplement: Supplementary file 5 — Additional file 5: Figure S5. The quality control of RNA-seq data sets and the classification of lncRNAs (take one of these samples for example). A sequencing data filtering; B sequencing error rate distribution; C GC content distribution; D genomic regional distribution. [file 13287_2023_3572_MOESM5_ESM.tif]

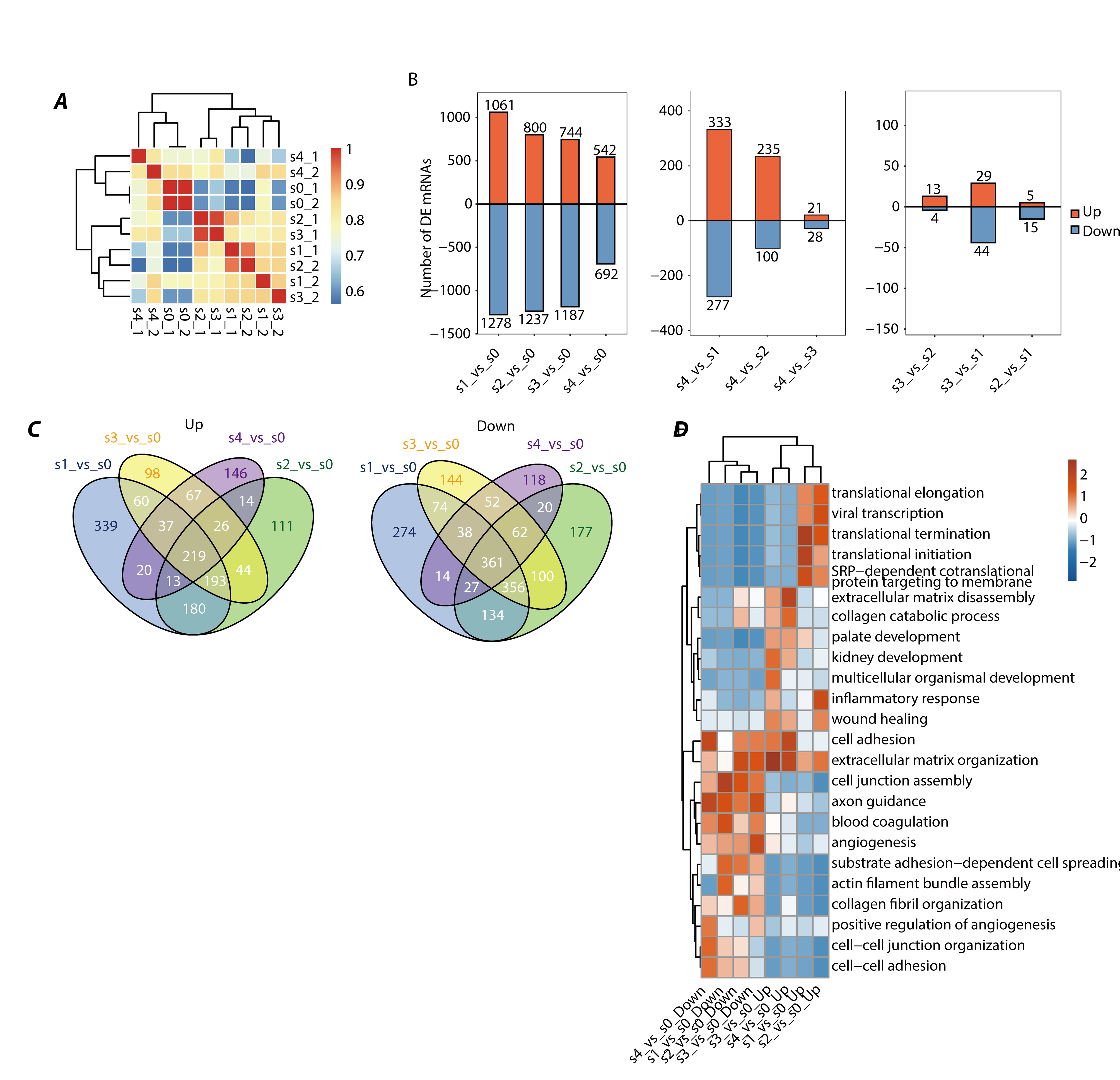

Supplement: Supplementary file 6 — Additional file 6: Figure S6. The expression of mRNAs at each stage and functional enrichment of differentially expressed mRNAs. A Heatmap clustering analysis of sample correlation based on the normalized mapped reads on each mRNAs; B bar plots of differential mRNAs at different stages; C the Venn diagram showed the overlap of differentially up-regulated or down-regulated mRNAs (S0 stage was compared with S1, S2, S3, and S4 stages, respectively); D S0 stage was compared with S1, S2, S3, and S4 stages, respectively, and GO (molecular process) enrichment analysis of differentially up-regulated or down-regulated mRNAs; the colour scale shows the significance of these terms scaled by column (-log10 corrected p value). [file 13287_2023_3572_MOESM6_ESM.tif]

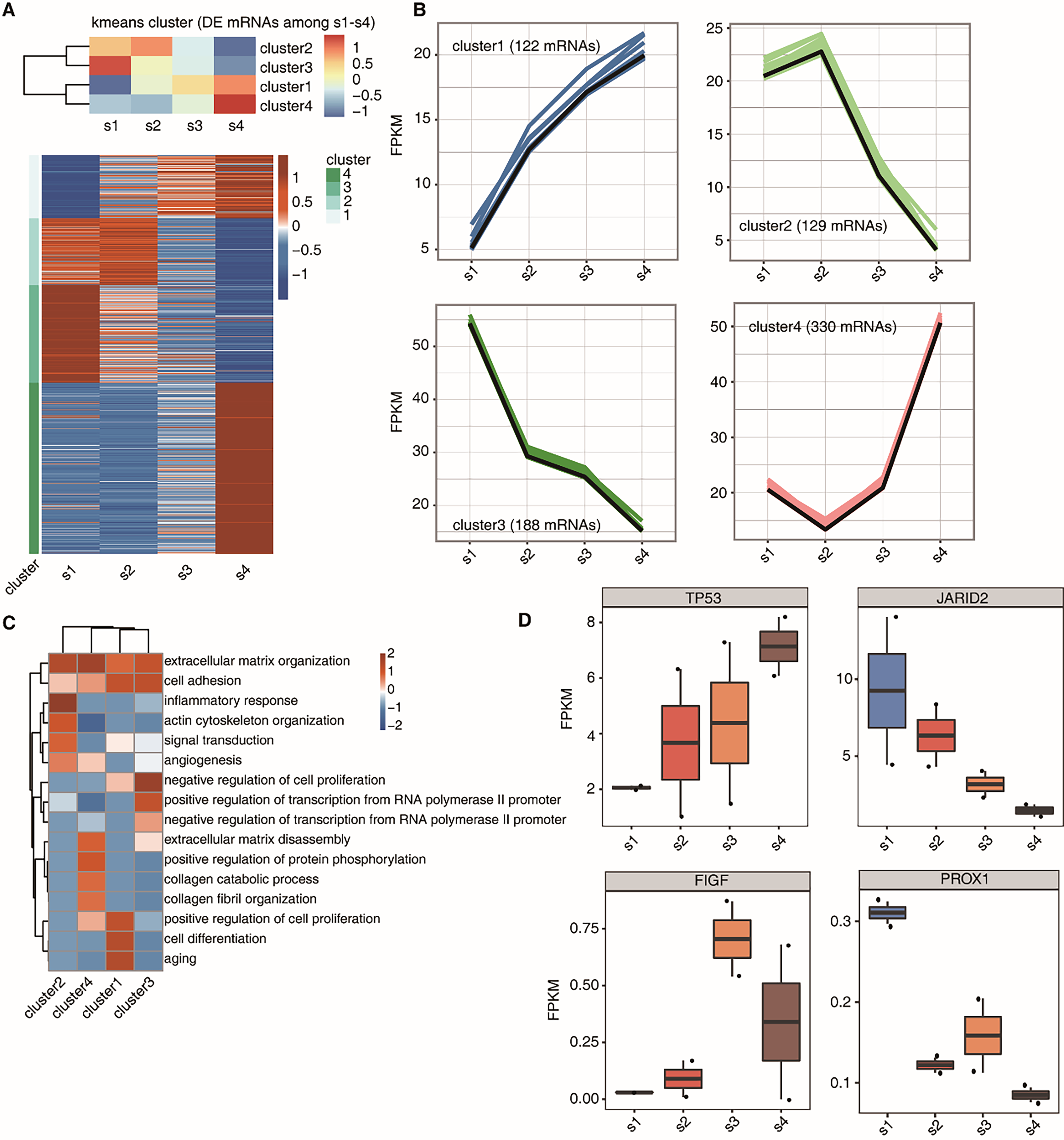

Supplement: Supplementary file 7 — Additional file 7: Figure S7. The expression pattern diagram of mRNAs in S1 stage to S4 stage was displayed by K-means analysis. A Differential mRNAs in S1 stage to S4 stage were clustered by K-means; B Gene expression profiles generated by K-means clustering; C the four clusters of differential mRNAs generated by K-means clustering showed the top five most enriched GO terms (molecular processes); the colour scale shows the significance of these terms by column (-log10 corrected p value); D the expression of four mRNAs at each stage. [file 13287_2023_3572_MOESM7_ESM.tif]

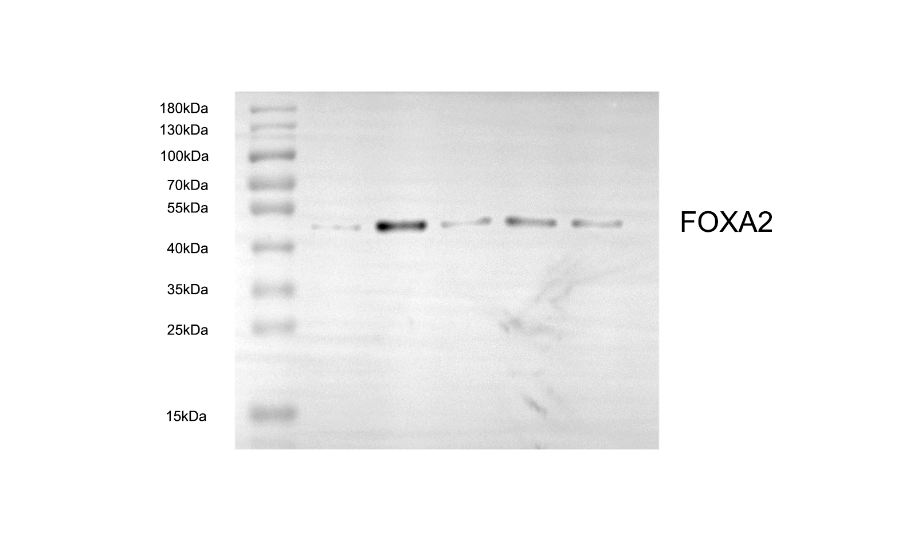


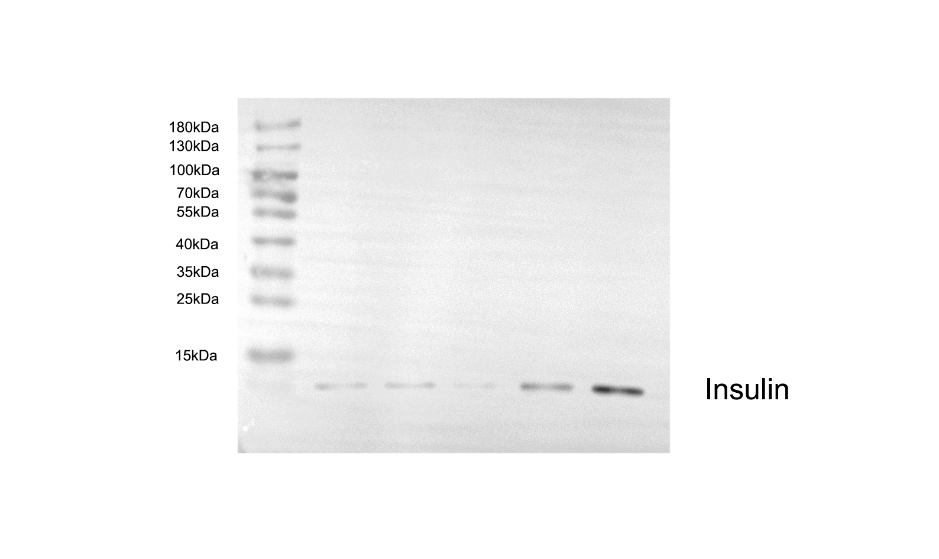

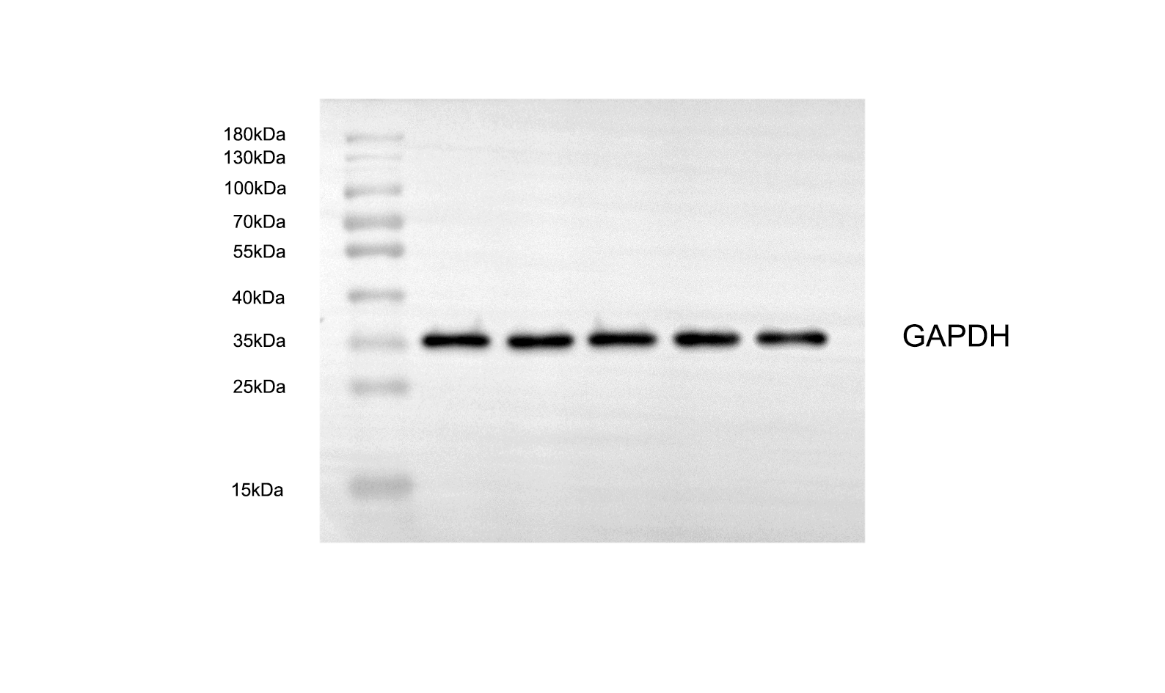

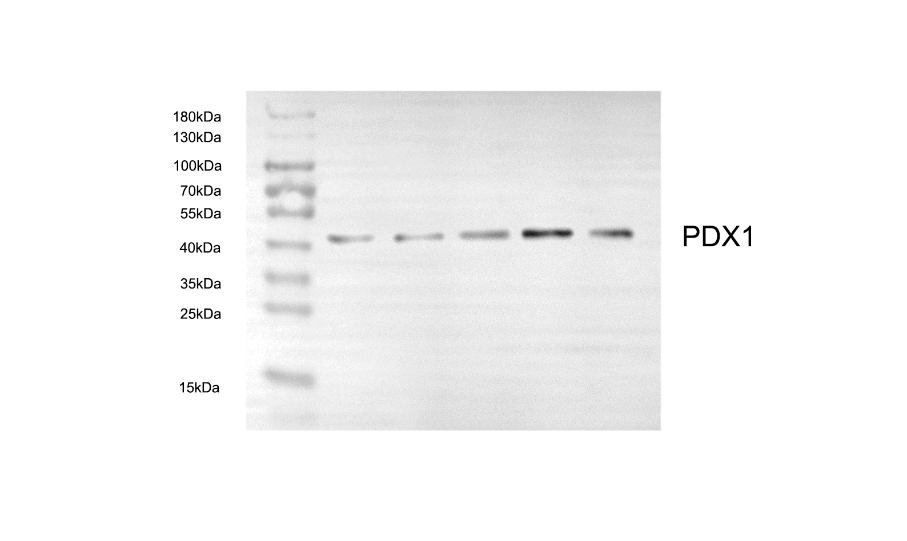


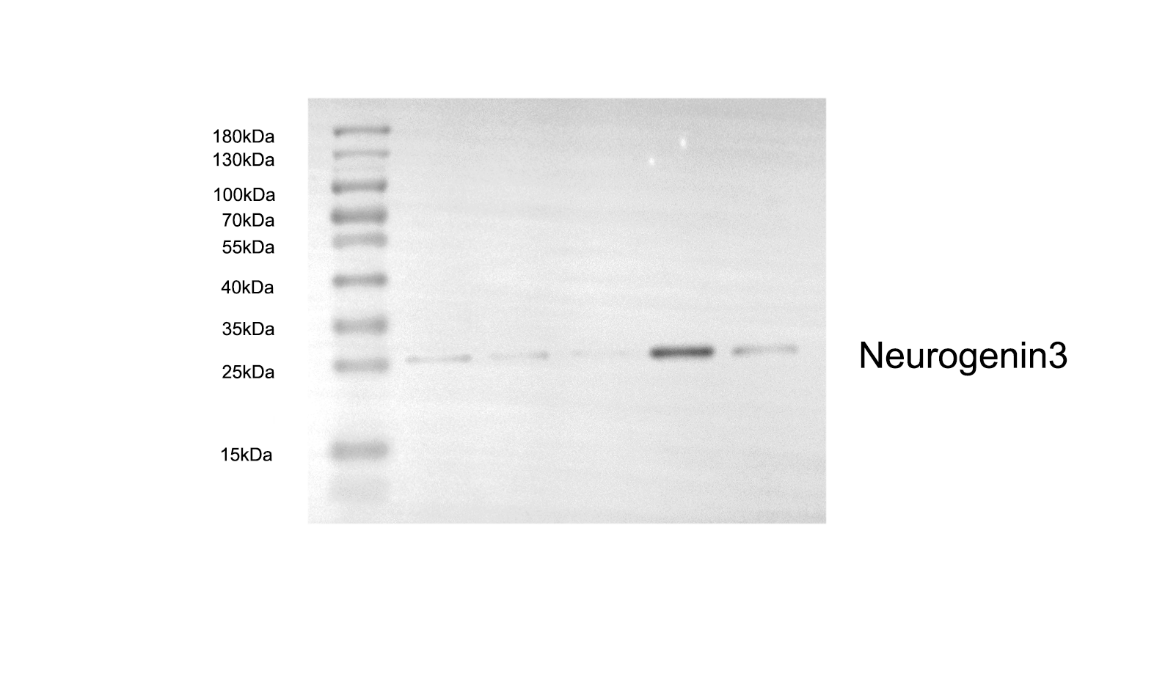

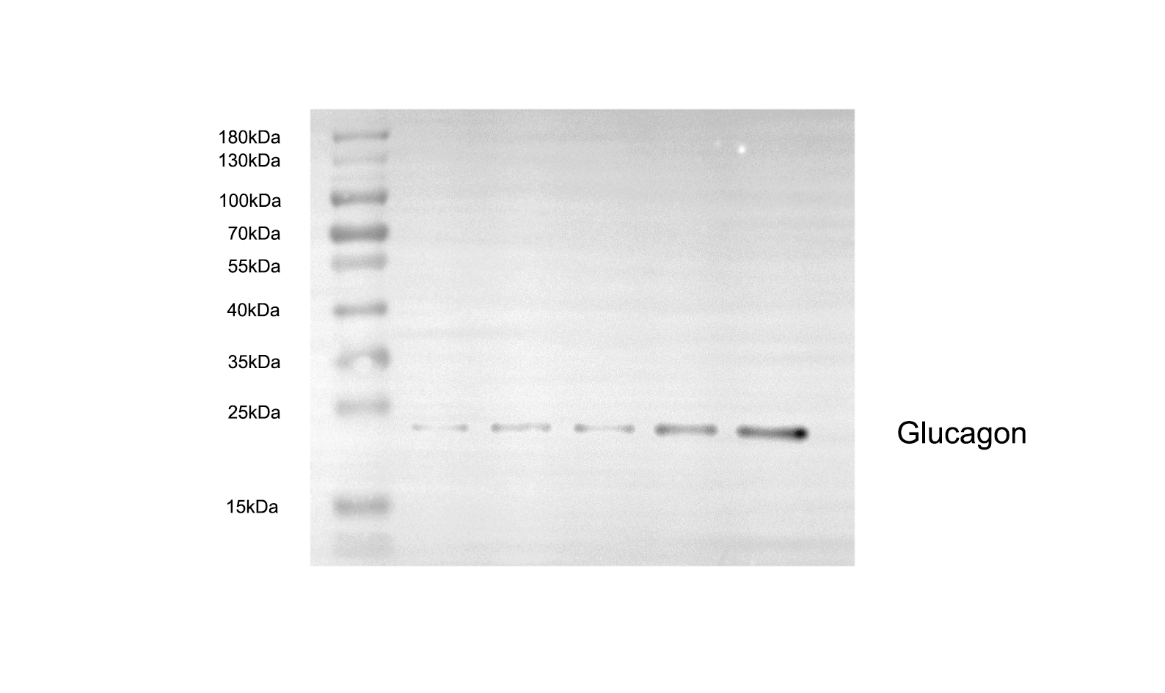

Supplement: Supplementary file 8 — Additional file 8: Original image of the protein in Fig. S2A. [file 13287_2023_3572_MOESM8_ESM.docx]
