# Supplementary material for: Dysregulated lncRNAs regulate human umbilical cord mesenchymal stem cell differentiation into insulin-producing cells by forming a regulatory network with mRNAs
Source: Stem Cell Res Ther. 2024 Jan 25;15:22. doi: 10.1186/s13287-023-03572-5 (PMC10809572; doi:10.1186/s13287-023-03572-5)
Supplement: Supplementary file 9 — Additional file 9: Table S1. The quality control of RNA-seq data sets. [file 13287_2023_3572_MOESM9_ESM.docx]

| Sample-name | Raw-reads | Clean_  reads | Error-rate(%) | GC-  content(%) | Total-mapped | Multiple- mapped | Uniquely mapped |
| --- | --- | --- | --- | --- | --- | --- | --- |
| s0_1 | 85566046 | 84383994 | 0.03 | 48.22 | 81892779 (97.05%) | 2430943 (2.88%) | 79461836 (94.17%) |
| s0_2 | 78391758 | 77285604 | 0.02 | 48.41 | 75160449 (97.25%) | 2182007 (2.82%) | 72978442 (94.43%) |
| s1_1 | 94749764 | 93146476 | 0.03 | 48.55 | 89465329 (96.05%) | 3369527 (3.62%) | 86095802 (92.43%) |
| s1_2 | 84186830 | 82864242 | 0.02 | 49.39 | 79702358 (96.18%) | 2879121 (3.47%) | 76823237 (92.71%) |
| s2_1 | 82018894 | 81254546 | 0.03 | 47.33 | 78599723 (96.73%) | 2406658 (2.96%) | 76193065 (93.77%) |
| s2_2 | 86445100 | 85287152 | 0.03 | 51.93 | 79548057 (93.27%) | 4756549 (5.58%) | 74791508 (87.69%) |
| s3_1 | 87287178 | 85746036 | 0.02 | 47.7 | 83089029 (96.9%) | 2565106 (2.99%) | 80523923 (93.91%) |
| s3_2 | 97429038 | 96550722 | 0.03 | 47.53 | 92659163 (95.97%) | 3006546 (3.11%) | 89652617 (92.86%) |
| s4_1 | 85833610 | 84795794 | 0.03 | 48.68 | 81996295 (96.7%) | 3259674 (3.84%) | 78736621 (92.85%) |
| s4_2 | 78592124 | 77954490 | 0.02 | 48.33 | 75235663 (96.51%) | 2492628 (3.2%) | 72743035 (93.31%) |

TableS1：**The quality control of RNA-seq data sets**
