# Supplementary material for: Dysregulated lncRNAs regulate human umbilical cord mesenchymal stem cell differentiation into insulin-producing cells by forming a regulatory network with mRNAs
Source: Stem Cell Res Ther. 2024 Jan 25;15:22. doi: 10.1186/s13287-023-03572-5 (PMC10809572; doi:10.1186/s13287-023-03572-5)
Supplement: Supplementary file 10 — Additional file 10: Table S2. Relevant antibody information for the Western blot. [file 13287_2023_3572_MOESM10_ESM.docx]

Supplemental tableS2

**Primary antibody**

| Primary antibody name | Brand | Item No. |
| --- | --- | --- |
| Foxa2 | Abcam | Ab108422 |
| Pdx1 | Abcam | Ab219207 |
| glucagon | Abcam | Ab92517 |
| Insulin | ABclonal | A2090 |
| Neurogenin3 | Bioss | bs-0922R |
| GAPDH | Abcam | Ab8245 |

**Secondary antibody**

| Secondary antibody name | Brand | Item No. |
| --- | --- | --- |
| HRP-Goat anti Rabbit | Elabscience | E-AB-1003 |
| HRP-Goat anti Mouse | Elabscience | E-AB-1001 |
